# Transcriptional regulation of *Satb1* in mouse trophoblast stem cells

**DOI:** 10.3389/fcell.2022.918235

**Published:** 2022-12-14

**Authors:** Wei Yu, V. Praveen Chakravarthi, Shaon Borosha, Iman Dilower, Eun Bee Lee, Anamika Ratri, Rebekah R. Starks, Patrick E. Fields, Michael W. Wolfe, M. Omar Faruque, Geetu Tuteja, M. A. Karim Rumi

**Affiliations:** ^1^ Department of Pathology and Laboratory Medicine, University of Kansas Medical Center, Kansas City, KS, United States; ^2^ Department of Genetics, Development and Cell Biology, Iowa State University, Ames, IA, United States; ^3^ Department of Cell Biology and Physiology, University of Kansas Medical Center, Kansas City, KS, United States

**Keywords:** SATB homeobox 1, trophoblast stem cells, transcriptional regulation, distant-acting enhancer, chromatin looping

## Abstract

SATB homeobox proteins are important regulators of developmental gene expression. Among the stem cell lineages that emerge during early embryonic development, trophoblast stem (TS) cells exhibit robust SATB expression. Both SATB1 and SATB2 act to maintain the trophoblast stem-state. However, the molecular mechanisms that regulate TS-specific *Satb* expression are not yet known. We identified Satb1 variant 2 as the predominant transcript in trophoblasts. Histone marks, and RNA polymerase II occupancy in TS cells indicated an active state of the promoter. A novel cis-regulatory region with active histone marks was identified ∼21 kbp upstream of the variant 2 promoter. CRISPR/Cas9 mediated disruption of this sequence decreased Satb1 expression in TS cells and chromosome conformation capture analysis confirmed looping of this distant regulatory region into the proximal promoter. Scanning position weight matrices across the enhancer predicted two ELF5 binding sites in close proximity to SATB1 sites, which were confirmed by chromatin immunoprecipitation. Knockdown of ELF5 downregulated Satb1 expression in TS cells and overexpression of ELF5 increased the enhancer-reporter activity. Interestingly, ELF5 interacts with SATB1 in TS cells, and the enhancer activity was upregulated following SATB overexpression. Our findings indicate that trophoblast-specific Satb1 expression is regulated by long-range chromatin looping of an enhancer that interacts with ELF5 and SATB proteins.

## 1 Introduction

SATB homeobox proteins (SATB1 and SATB2) are global chromatin organizers and transcriptional regulators important for tissue specific gene expression and cell lineage development. SATB proteins bind to AT-rich elements in matrix-attachment regions of actively transcribing DNA and interact with chromatin remodeling proteins as well as transcription factors to activate or repress gene expression (Dickinson et al., 1992; Dickinson et al., 1997; Yasui et al., 2002; Cai et al., 2003; Dobreva et al., 2003; Cai et al., 2006). SATB proteins play key roles in developmental processes, such as T cell differentiation (Alvarez et al., 2000; Nakayama et al., 2005; Notani et al., 2010), erythroid development (Wen et al., 2005), osteoblast differentiation and craniofacial patterning (Dobreva et al., 2006), cortical neuron organization (Alcamo et al., 2008; Britanova et al., 2008; Gyorgy et al., 2008), hematopoietic stem cell self-renewal (Will et al., 2013), and embryonic stem (ES) cell pluripotency (Savarese et al., 2009). A recent study has reported that SATB proteins play distinct roles in lineage determination during early embryonic development (Goolam and Zernicka-Goetz, 2017). In our previous studies, we demonstrated that SATB proteins act to maintain the trophoblast cell stem-state and inhibit trophoblast differentiation (Kent et al., 2010; Asanoma et al., 2012).

SATB proteins are expressed abundantly in both mouse and rat trophoblast stem (TS) cells while in the stem-state, but the expression declines during differentiation (Kent et al., 2010; Asanoma et al., 2012). During early gestation, trophoblast cells also show high levels of SATB expression, which decreases with the progression of gestation (Kent et al., 2010; Asanoma et al., 2012). Differential expression in the trophoblast stem-state indicates a potential role for TS-specific transcriptional regulators in controlling *Satb1* expression. However, the mechanisms responsible for regulating *Satb1* gene expression in TS cells or in the placenta are currently unknown.

SATB proteins are important regulators of TS cell renewal and differentiation (Asanoma et al., 2012). TS cells are the precursors of specialized differentiated cell types in the placenta. Self-renewal of TS cells and regulated differentiation into multiple trophoblast lineages are essential for proper placental development and function, as well as maintenance of pregnancy (Cockburn and Rossant, 2010; Roberts and Fisher, 2011; Pfeffer and Pearton, 2012). These proteins are a part of a regulatory network that controls the development of the trophoblast lineage and regulates its differentiation. Insight into the transcriptional regulation of SATB expression in trophoblast cells will provide opportunities to manipulate its expression, which could have a wide range of applications in experimental biology.

In this study, we detected *Satb1* transcript variants that are preferentially expressed in trophoblast cells and identified their promoters. We also identified a distant-acting *cis* enhancer that forms long-range chromatin interactions with the proximal promoters to regulate trophoblast-specific *Satb1* expression.

## 2 Materials and methods

### 2.1 Cell culture

Two TS cell models were included in this study: mouse TS cells and Rcho1 rat TS cells. Mouse TS cells (obtained from Dr. Janet Rossant, Hospital for Sick Children, Toronto, Canada) were maintained in FGF4/heparin supplemented TS culture medium [containing 30% TS basal medium (RPMI supplemented with 20% FBS, 1 mM sodium pyruvate and 100 μM 2-mercaptoethanol), 70% mouse embryonic fibroblast-conditioned medium, 25 ng/ml FGF4 and 1 μg/ml heparin] as described previously (Tanaka et al., 1998). Differentiation of the cells was induced by removal of FGF4, heparin and mouse embryonic fibroblast conditioned medium (Tanaka et al., 1998). ES-E14Tg2A (E14) mouse embryonic stem (ES) cells (obtained from ATCC, Manassas, VA) were maintained in RESGRO (SCM001) culture media (EMD Millipore) on feeder-free, gelatin-coated culture dishes. Extraembryonic endoderm stem (XEN) cells (obtained from Dr. Janet Rossant) were grown in Base XEN medium (RPMI supplemented with 15% FBS, 1 mM sodium pyruvate and 50 μM 2-mercaptoethanol) as published earlier (Kunath et al., 2005). Rcho-1 TS cells (a rat choriocarcinoma cell line obtained from Dr. Michael Soares, University of Kansas Medical Center, Kansas City, KS) were maintained in TS basal medium (RPMI supplemented with 20% FBS, 1 mM sodium pyruvate and 50 μM 2-mercaptoethanol), as previously reported (Sahgal et al., 2006). Differentiation was induced by growing the cells to near confluence and removing FBS (Sahgal et al., 2006). 293FT cells (purchased from Thermo Fisher Scientific) were maintained in DMEM supplemented with 10% FBS and 4 mM glutamine. All cell cultures were carried out at 37°C in a humidified 5% CO_2_ atmosphere.

To reprogram ES cells, pCAG-hCdx2ERT2-ires-puro (obtained from Dr. Jon Draper, McMaster University, Canada) or pCAG-hGata3ERT2-ires-puro (obtained from Dr. Janet Rossant) vectors were stably transfected into E14 mouse ES cells using lipofectamine 2000 (Thermo Fisher scientific). Cells were selected for puromycin resistance, and transgenes were activated by supplementing TS medium with 1 μg/ml 4-OH tamoxifen (Millipore Sigma). Cells were fed daily with the tamoxifen containing TS medium for 6 days and analyzed for gene expression as described in a previous publication (Ralston et al., 2010). Human ES cells (ESI-017, ESI BIO, Alameda, CA) were converted to trophoblasts by exposing them to BAP (BMP4, A83-01 and PD173074) in the absence of FGF2 for 2 days and analyzed for gene expression as described previously (Amita et al., 2013).

### 2.2 Gene expression analysis

Gene expression analysis at the mRNA level was performed by conventional RT-PCR, RT-qPCR, and RNA-seq, whereas cellular protein expression was assessed by immunofluorescence and western blot analysis.

#### 2.2.1 RT-PCR and qRT-PCR

RNA was extracted by using TRI Reagent (Sigma-Aldrich) according to manufacturer’s instructions. cDNAs were reverse transcribed from 2 μg of total RNA by using Applied Biosystems High-Capacity cDNA Reverse Transcription Kits (Thermo Fisher Scientific). Conventional PCR amplification of cDNA was done in a 25 μl reaction volume by using DreamTaq Green DNA polymerase (Thermo Fisher Scientific). Real-time RT-qPCR amplification of cDNAs was carried out in a 20 μl reaction mixture containing Applied Biosystems Power SYBR Green PCR Master Mix (Thermo Fisher Scientific). Amplification and fluorescence detection of qRT-PCR were carried out on Applied Biosystems StepOne Real Time PCR System (Thermo Fisher Scientific). The ΔΔCT method was used for relative quantification of target mRNA normalized to 18S RNA. All PCR primers were designed using Primer3 (Untergasser et al., 2007) and the sequences are shown in Supplementary Table S1–S3.

#### 2.2.2 RNA sequencing

RNA-Seq data was previously generated and analyzed (Tuteja et al., 2016). FPKM values were extracted from data deposited in GEO, under accession GSE65808.

#### 2.2.3 Immunofluorescent Microscopy

Mouse ES, TS or XEN cells were grown on coverslips placed in six-well tissue culture plates. After fixation in 4% formaldehyde for 10 min and permeabilization in 0.5% Triton X-100 for 10 min, the coverslips were blocked with 5% BSA for 1 h at room temperature. After blocking, the cells were incubated with appropriately diluted primary antibodies: anti-SATB1 (ab109122, Abcam at 1:1,000) and either anti-CDX2 (cdx2-88, BioGenex at 1:200), or anti-OCT4 (Sc-5279, Santa Cruz Biotechnology at 1:200) or anti-GATA4 (sc-25310, Santa Cruz Biotechnology at 1:200) at room temperature for 2 h. After washing the unbound primary antibodies, secondary antibody staining was performed with Alexa Fluor 568- or 488- labeled detection reagents (goat anti-rabbit, goat anti-mouse antibodies; Molecular Probes) at 1:200 dilution, and DNA staining was performed by DAPI (Prolong Gold Antifade Mountant, Thermo Fisher Scientific). The images were captured on a Nikon Eclipse 80i microscope.

#### 2.2.4 Western Blotting

Cell lysates were prepared in 1x SDS Sample Buffer (62.5 mM Tris-HCl pH 6.8, 2%SDS, 42 mM DTT, 10% glycerol and 0.01% bromophenol blue; Cell Signaling Technology), sonicated to shear DNA and reduce viscosity and then heat denatured. Proteins were separated on 4%–20% SDS-PAGE and transferred to PVDF membranes. Membranes were blocked with 5% milk and incubated with primary antibodies for 1 h at room temperature. Then the membranes were incubated with following primary antibodies at appropriate dilution in blocking buffer: ant-SATB1 (ab109122, Abcam 1: 10,000), anti-SATB2 (ab92446, sc-81376, 1:2000), anti-CDX2 (Abcam, 1:5,000), anti-OCT4 (sc-5279, Santa Cruz Biotechnology, 1:2,000), anti-GATA4 (sc-25310, Santa Cruz Biotechnology, 1:2,000), anti-FLAG (#14793, Cell Signaling Technology, 1:5,000) and ELF5 (sc-9645, Santa Cruz Biotechnology, 1:2,000). Anti-TUBA (MABT522, Millipore Sigma, 1:20,000), anti-ACTB (A5441, Millipore Sigma, 1:30,000) or anti-Histone H3 (ab1791, Abcam, 1:20,000) antibodies were used detect the expression of housekeeping genes as loading controls. Membranes were washed, blocked, and incubated with peroxidase-conjugated anti-mouse, anti-rabbit, or anti-goat secondary antibodies (Santa Cruz Biotechnology) at a dilution of 1:5,000–2,0000, and immunoreactive signals were visualized using Luminata Crescendo Western HRP substrate (Millipore Sigma).

### 2.3 Analysis of transcriptional landscape in *Satb1* promoter and enhancer

Trophoblast-specific *Satb1* promoters were initially located by variant specific RT-PCR and RNA sequencing as described above. The locations of the proximal promoters and the distant-acting *Satb1* enhancer were identified by analyses of H3K27ac ChIP-seq data. Identified promoters and the enhancer were further characterized for relevant histone marks and transcription factor binding by ChIP analyses.

#### 2.3.1 ChIP-Seq analyses for H3K27ac in mouse early placentas

ChIP-Seq data was previously generated and analyzed (Tuteja et al., 2016). Peak data was downloaded from GEO (GSE65807). Normalized wiggle signal tracks were generated using the bam_to_bigwig function in pybedtools (Dale et al., 2011).

#### 2.3.2 Chromatin Immunoprecipitation of mouse TS and Rcho1 rat TS cells

Each ChIP sample was prepared with 15–20 million mouse TS, or Rcho1 rat TS cells as described earlier (Chuong et al., 2013). Briefly, cells were cross-linked in 1% formaldehyde for 10 min at room temperature, quenched in 0.125 M glycine for 5 min, washed twice with cold PBS with 0.5% IGEPAL CA-630 and resuspended in cold lysis buffer (50 mM Tris-HCl, pH 8, 10 mM EDTA, 0.2% SDS) in the presence of PMSF and protease inhibitor cocktail (Sigma-Aldrich) for 30 min. Cell lysates were diluted 1:1 with dilution buffer (0.01% SDS, 1.1% Triton X-100,1.2 mM EDTA, 16.7 mM Tris-HCl, pH 8.1, 167 mM NaCl) then sonicated for 40 cycles (20 s on/60 s off) at 70% amplitude to produce an average fragment size range of 300 bp–600 bp. Immunoprecipitation was performed using ∼2.5 µg–5 µg antibody (anti-H3K27ac: 05–1,334 Millipore Sigma, anti-H3K9ac: 07–352 Millipore Sigma, anti-H3K4me3: 07–473 Millipore Sigma, anti-SATB1: ab109122 Abcam, anti-SATB2: sc-81376 Santa Cruz Biotechnology, anti-ELF5: sc-9645x Santa Cruz Biotechnology, anti-Pol II: sc-47701 Santa Cruz Biotechnology, anti-FLAG M8823 Millipore Sigma) conjugated to 50 µl protein A/G magnetic beads (Dynabeads, Thermo Fisher Scientific) overnight. Bead-chromatin complexes were washed using High Salt Buffer (0.1% SDS, 1%Triton X-100, 2 mM EDTA, 20 mM Tris-HCl, pH 8.1, 500 mM NaCl), Low Salt Buffer (0.1% SDS, 1% Triton X-100, 2 mM EDTA, 20 mM Tris-HCl, pH 8.1, 150 mM NaCl), LiCl Buffer (0.25 M LiCl, 1% IGEPAL, 1% Deoxycholic acid, 1 mM EDTA, 10 mM Tris-HCl, pH 8.1) and TE buffer (10 mM Tris-HCl, 1 mM EDTA, pH 8.0), with each wash performed twice for 5 min. Cell lysis, sonication, immunoprecipitation, and cleanup steps were all performed at 4°C. Finally, chromatin DNA was eluted from the magnetic beads using elution buffer (1% SDS, 0.1 M NaHCO3), protein-DNA crosslinks were reversed with the addition of 5 M NaCl and heating on a shaker incubator overnight and purified using Qiaquick columns (Qiagen). DNA was eluted in 100 µl of 10 mM Tris-HCl and 2.5 µl–5 µl aliquots were used in qPCR analyses. qPCR primers for the target sites are shown in Supplementary Table S4. Mouse positive control primer set Actb2 (#71017, Active Motif) and mouse negative control primer set 1 (#71011, Active Motif) were used for validating the ChIP assays (Supplementary Figure S2C–F).

### 2.4 Characterization of the distant-acting Satb1 enhancer

Requirement of the distant-acting enhancer in transcriptional regulation of *Satb1* was assessed by targeted disruption of the locus using CRISPR/Cas9. Chromatin looping and interaction of the distant enhancer with the proximal promoter was demonstrated by chromosome conformation capture (3C).

#### 2.4.1 CRISPR/Cas9 mediated interference and deletion of the enhancer

CRISPR guide RNAs that specifically target the *Satb1* var2 promoter and *enhancer S* were designed to have limited off-targets using an online tool (http://crispr.mit.edu/). All gRNA sequences are listed in SupplementaryTable S5. Oligonucleotides encoding the gRNAs were annealed and cloned into the phU6-gRNA (Addgene, Plasmid #53188) (Kabadi & Gersbach, 2014) following guidelines from the Zhang lab (http://www.genome-engineering.org/crispr/? page_id=23). Rcho1 TS cells, a commonly used rat TS cell model, was selected for the CRISPR/Cas9 mediated targeted deletion experiments because of its high transfection efficiency. For CRISPR/Cas9 mediated targeted deletion of the enhancer, Rcho1 cells were stably cotransfected with the vectors (phU6-gRNA) expressing enhancer gRNAs and Cas9 (pLV hUbc-Cas9-T2A-GFP, Addgene, Plasmid #53190) (Kabadi & Gersbach, 2014) using Lipofectamine 2,000 transfection reagent (ThermoFisher Scientific) and selected for G418 resistance and GFP expression. Selected cells were screened for targeted deletion of *Satb1* enhancer (Δ Enh S) using the PCR primers in SupplementaryTable S6 and characterized for trophoblast stem and differentiation markers. For CRISPR-interference, Rcho1 cells were co-transfected with the gRNA and dCas9 expression vector (pLV hUbc-dCas9-T2A-GFP; Addgene, Plasmid #53191) (Ralston et al., 2010). After 3 days of transfection, cells were harvested for RNA isolation and analyses of *Satb1* expression.

#### 2.4.2 Chromosome conformation capture

3C was carried out following a standard protocol (Dekker et al., 2002). 3C experiments performed in mouse TS cells were compared with that in mouse embryonic fibroblasts that do not express *Satb1*. Briefly, mouse TS cells and mouse embryonic fibroblasts were fixed in 1% formaldehyde for 10 min at room temperature. After quenching the crosslinking reaction with 0.125 M glycine for 5 min, cells were washed with cold PBS, resuspended in cold lysis buffer (10 mM Tris-HCl pH 7.5, 10 mM NaCl, 5 mM MgCl2, 0.1 mM EGTA with protease inhibitors) and incubated on ice for 30 min. After centrifugation at 2,000 *g* for 5 min, pelleted nuclei were resuspended in 2 ml of cold lysis buffer. Approximately 10^7^ nuclei were resuspended in 500 μl of 1.2x FastDigest Restriction Enzyme Buffer (Thermo Fisher Scientific) containing 1.6% SDS and incubated for 1 h at 37°C with shaking at 250 rpm. SDS was subsequently quenched by adjusting the reaction to 2% Triton-X100 followed by another 1 h incubation at 37°C with shaking. An aliquot of 20 μl was taken from each sample and stored at −20°C for use as undigested genomic DNA. Then 50 μl of FastDigest *Bgl* II restriction enzyme (Thermo Fisher Scientific) was added to the reaction tube and incubated overnight at 37°C with shaking at 250 rpm. The restriction enzyme was deactivated by adding 40 μl of 20% SDS and heating at 65°C for 20 min. The reaction was diluted in 7 ml of 1.1x T4 DNA ligase reaction buffer (Thermo Fisher Scientific), and 375 μl of 20% Triton-X100 was added and incubated at 37°C for 1 h to quench SDS. Digested chromatin was ligated with 150U of T4 DNA ligase (Thermo Fisher Scientific) for 4 h at 16^°^C. Formaldehyde crosslinks were reversed with Proteinase K digestion and overnight incubation at 65^°^C. RNAs were degraded with RNase treatment at 37^°^C for 1 h. 3C libraries were purified by phenol-chloroform extraction and precipitated with 2.5 volumes of 100% ethanol and 0.1 volume of 3 M sodium acetate and incubating at −80°C for 1 h. Precipitated DNA was collected by centrifugation at 5,000 *g* for 1 h and washed in 70% ethanol. DNA pellets were resuspended in 150 µl of 10 mM Tris-HCl pH 7.5 and 3C products were checked by conventional PCR. PCR primers used in 3C analysis are shown in SupplementaryTable S7.

### 2.5 Transcription factor binding to the distal enhancer

Putative ELF5 and SATB1 binding sites were identified in the *Satb1* enhancer (chr17: 51993298–51994604) using TFBSTools (Tan & Lenhard, 2016), and a 90% match threshold. Position weight matrices (PWMs) for ELF5 and SATB1 were obtained from a motif library described previously (Wenger et al., 2013). This analysis predicted multiple ELF5 binding sites near SATB1 binding sites. Further confirmation of these potential transcription factor binding sites was done by enhancer-reporter luciferase assays, ChIP analyses and investigating a possible interaction between ELF5 and SATB1.

#### 2.5.1 Luciferase reporter assays

To prepare the enhancer-reporter constructs, the *Satb1* enhancer sequence was cloned into the *Kpn*I and *Xho*I sites of pGL4.25 [luc2CP/minP] firefly luciferase vector containing a minimal TATA promoter (Promega). Rcho1 TS cells were used for the reporter assay. Twenty-four h after plating in 12-well plates, Rcho1 cells were transfected with the enhancer-reporter vector along with a control Renilla luciferase vector (pGL4.74 [hRluc/TK]) using Lipofectamine 2,000 (Thermo Fisher Scientific). Expression vectors for SATB1, SATB2 or ELF5 were individually cotransfected with the reporter vector to assess their regulatory role on the enhancer sequence. 12 h after the transfection, transfection medium was replaced with cell proliferation medium and cultured for another 12 h. 24 h after transfection, cells were washed with cold PBS, lysed in 100 µl of passive lysis buffer and standard dual luciferase assays were performed on the cell lysates by using Dual-Luciferase Reporter Assay reagents (Promega).

*ChIP assays*- ChIP assays were performed as described above.

#### 2.5.2 ELF5-SATB1 interaction

Protein-protein interaction was investigated by co-immunoprecipitation. Rcho1 cells stably expressing FLAG-tagged SATB1 or ELF5 were harvested to extract nuclear proteins. Nuclear proteins were extracted in nondenaturing buffer (20 mM Tris-HCl pH 7.5, 2 mM EDTA) adjusted to 0.3 M NaCl and 0.5% Triton X-100. After centrifugation at 40,000 g for 1 h at 4C in a Ti-70 rotor, the supernatants were mixed with anti-FLAG (M2) magnetic beads (Millipore Sigma) at a ratio of 100 μl of beads/1 ml of nuclear extract and gently rocked overnight at 40C. The beads with immunoprecipitated protein complexes were washed 8 times with wash buffer containing 50 mM Hepes-NaOH, pH 7.9, 0.25 M KCl, 0.1% Triton X-100, and then eluted with 200 μl of wash buffer containing 0.4 mg/ml FLAG peptide (Millipore Sigma). Eluted proteins were mixed with 2xSDS sample buffer, boiled for 10min, separated on SDS-PAGE, and processed for Western blot analysis.

#### 2.5.3 ELF5 regulation of *Satb1* expression in TS cells

The TS regulators ELF5 and SATB proteins demonstrated a high level of transcriptional activation of the *Satb1* enhancer in luciferase assays. We further analyzed the role of ELF5 in regulating *Satb1* expression using a ‘loss of function’ study.

#### 2.5.4 Elf5 knockdown

For the loss of function studies, *Elf5* was knocked down in Rcho1 cells by lentiviral delivery of shRNAs. *Elf5* shRNAs, cloned into the lentiviral vector pLKO.1, were obtained from Millipore Sigma (St. Louis, MO). A control shRNA that does not target any known mammalian gene, pLKO.1-shSCR (Addgene, Plasmid #1864), was obtained from Addgene (Cambridge, MA). Lentiviral packaging vectors from Addgene (pMDLg/pRRE Plasmid # 12251, pRSV-Rev Plasmid #12253 and pMD2. G Plasmid# 12259) were used to produce the viral particles in 293T cells as described earlier (Lee et al., 2009). Culture supernatants containing lentiviral particles were harvested every 24 h for 2 days, centrifuged to remove cell debris, filtered, and applied to Rcho1 cells in culture. Transduced cells were selected for puromycin resistance. *Elf5* knockdown as well as the effect of *Elf5* knockdown on *Satb1* expression was assessed by RT-qPCR assays. Functionally active shRNA sequences are shown in SupplementaryTable S8.

## 3 Results

### 3.1 Trophoblast-specific expression of *Satb1*

**FIGURE 1:**
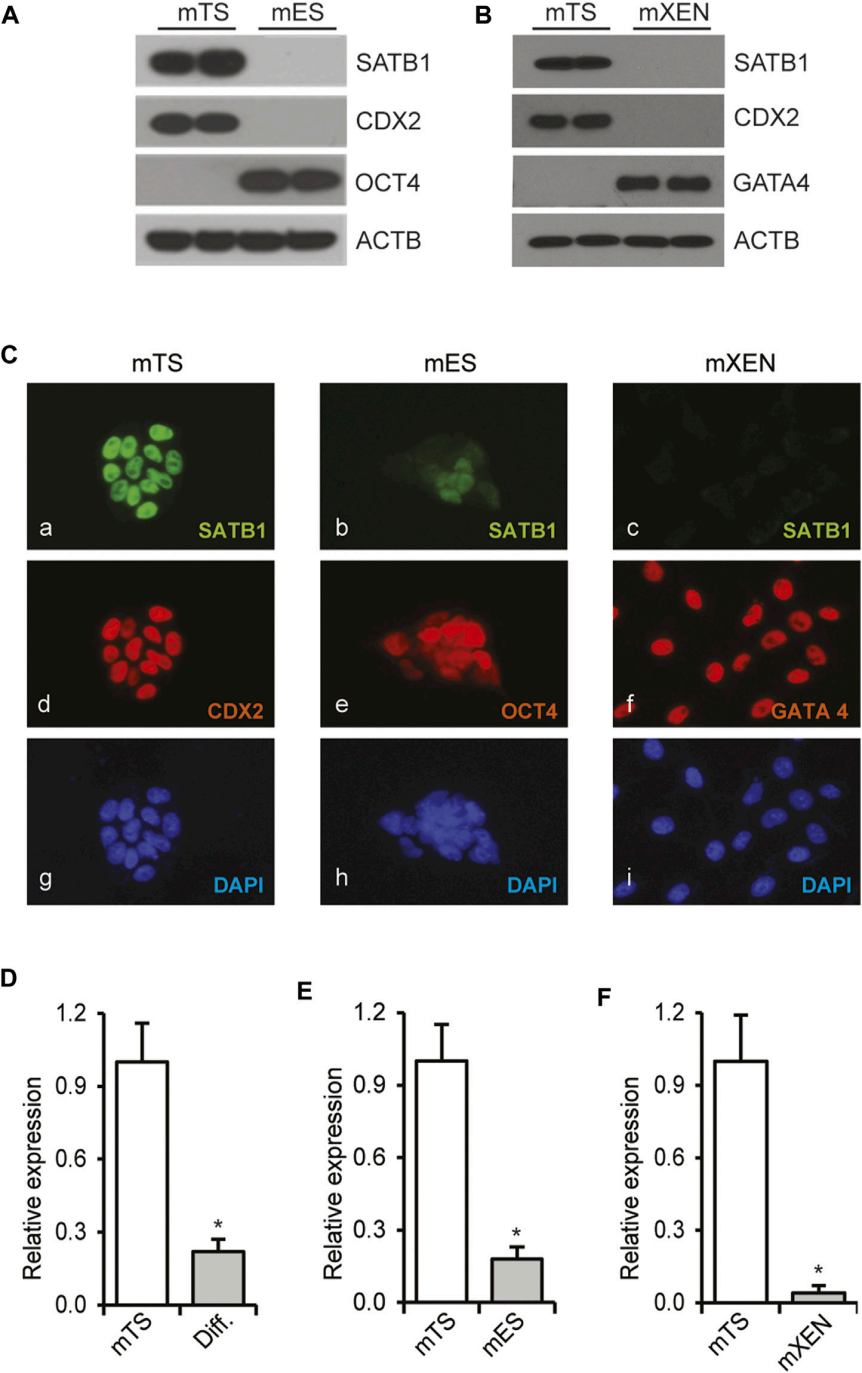
Trophoblast-specific expression of *Satb1*. Mouse trophoblast stem (mTS) cells express high levels of SATB1 compared to mouse embryonic stem (mES) cells **(A)** or mouse extraembryonic endoderm (mXEN) cells **(B)** as detected by western blotting. CDX2, OCT4, and GATA4 were used as lineage markers. ACTB was used as a loading control. Immunofluorescence imaging **(C)** demonstrated abundant expression of SATB1 in mTS cells (Ca). Compared to mTS cells, the level of expression is remarkably lower in mES cells (Cb) and mXEN (Cc). *Satb1* mRNA levels in TS, ES and XEN cells **(D–F)** correlated well with protein expression **(A–C)**, and in mTS cells, the mRNA levels were significantly reduced upon induction of trophoblast differentiation **(D)**. RT-qPCR data are expressed as mean ± S.D. *, *p* < 0.05 (*n* = 3). Diff. Differentiated mTS cells.

Expression of *Satb1* mRNA and protein was examined in mouse TS, ES and XEN cells. While mouse TS cells expressed robust levels of SATB1 as detected in Western blot or Immunofluorescence imaging analyses, the protein was not detected or barely detected in either mouse ES or XEN cells by either measure (Figures 1A–C). Expression of *Satb1* in mouse TS cells declined upon induction of trophoblast differentiation (Figure 1D). Mouse ES or XEN cells minimally express *Satb1* in the stem-state (Figures 1A–C,E,F); however, expression of *Satb1* was induced when mouse ES cells were reprogrammed to a trophoblast fate by overexpression of CDX2 (Supplementary FigureS1A) or GATA3 (Supplementary FigureS1D). Likewise, *SATB1* expression was also increased when human ES cells were differentiated into trophoblast cells following BMP4 treatment (Supplementary FigureS1G).

### 3.2 *Satb1* promoters in trophoblast cells

**FIGURE 2:**
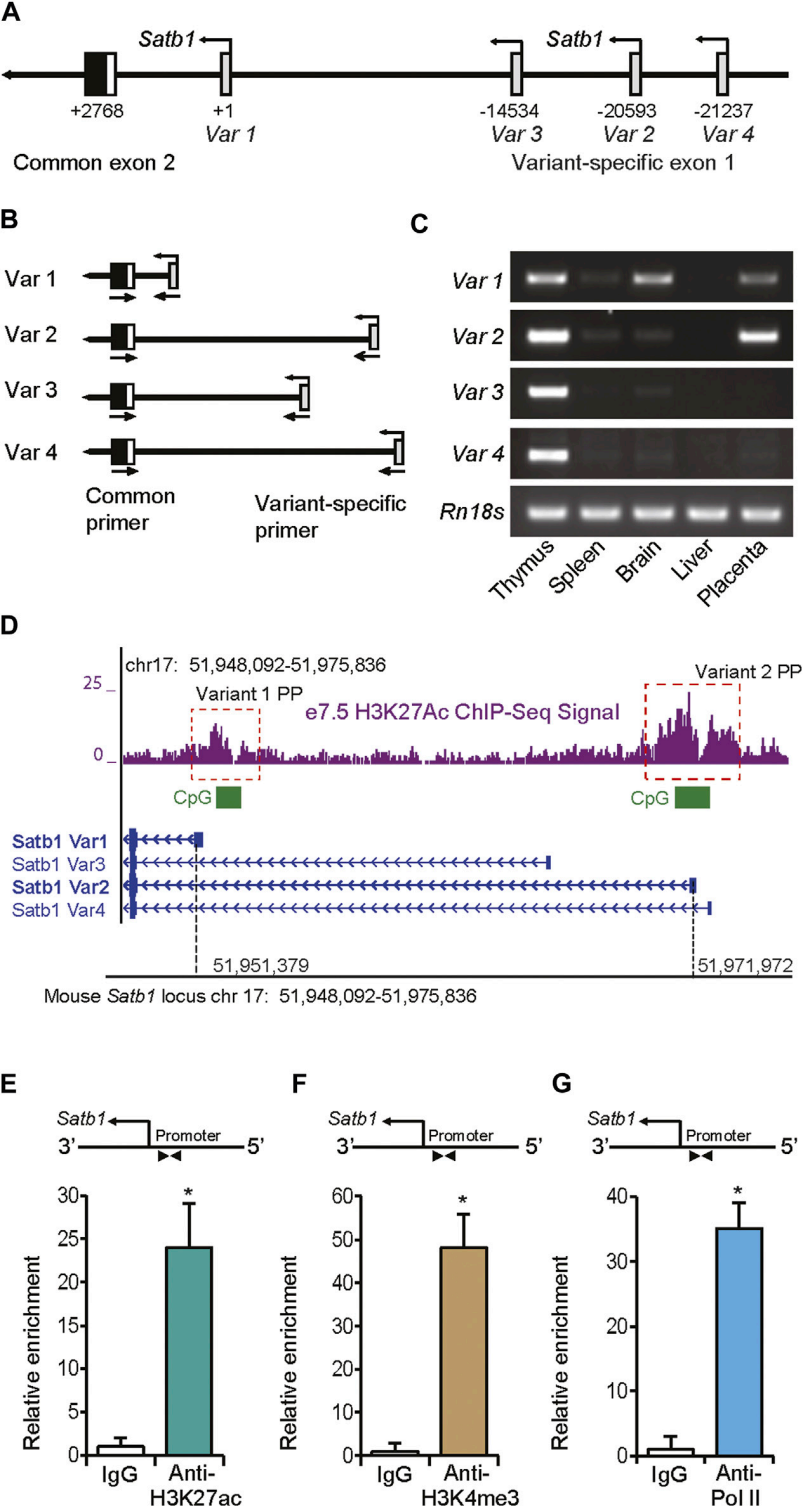
Detection of trophoblast-specific *Satb1* promoters. **(A)** Schematic presentation of the mouse *Satb1* gene locus showing four transcript variants, each transcribed from a variant-specific alternative exon 1. Nucleotide positions are indicated with respect to the start site (TSS) of variant 1. **(B)** Strategy of PCR-based detection of different transcript variants. **(C)** *Satb1* transcript variants and alternative transcription start sites were detected in mouse embryonic day 7.5 (e7.5) ectoplacental cone (EPC) by RT-PCR analyses. *Satb1* transcript variants in mouse thymus, spleen, brain, and liver were detected as controls for comparison. **(D)** ChIP-seq data on e7.5 EPCs demonstrated that both variant 1 and 2 proximal promoters possessed active histone marks of acetylated histone H3 lysine 27 (H3K27ac). The promoters also contained CpG islands **(D)**. Using ChIP assays, the variant 2 promoter in mouse TS cells was assessed for transcriptionally active histone marks, H3K27ac **(E)** and H3K4me3 **(F)**, which were associated with enriched RNA polymerase II (Pol II) binding **(G)**. ChIP-qPCR primers located in the proximal promoter region are shown schematically in **(E–G)**. The primer sequences are mentioned in Supplementary Table S4. ChIP-qPCR data are expressed as means ± S.D. *p < 0.05 (*n* = 3). Variant 1PP, Variant 1 proximal promoter, Variant 2PP, Variant 2 proximal promoter.

**FIGURE 3:**
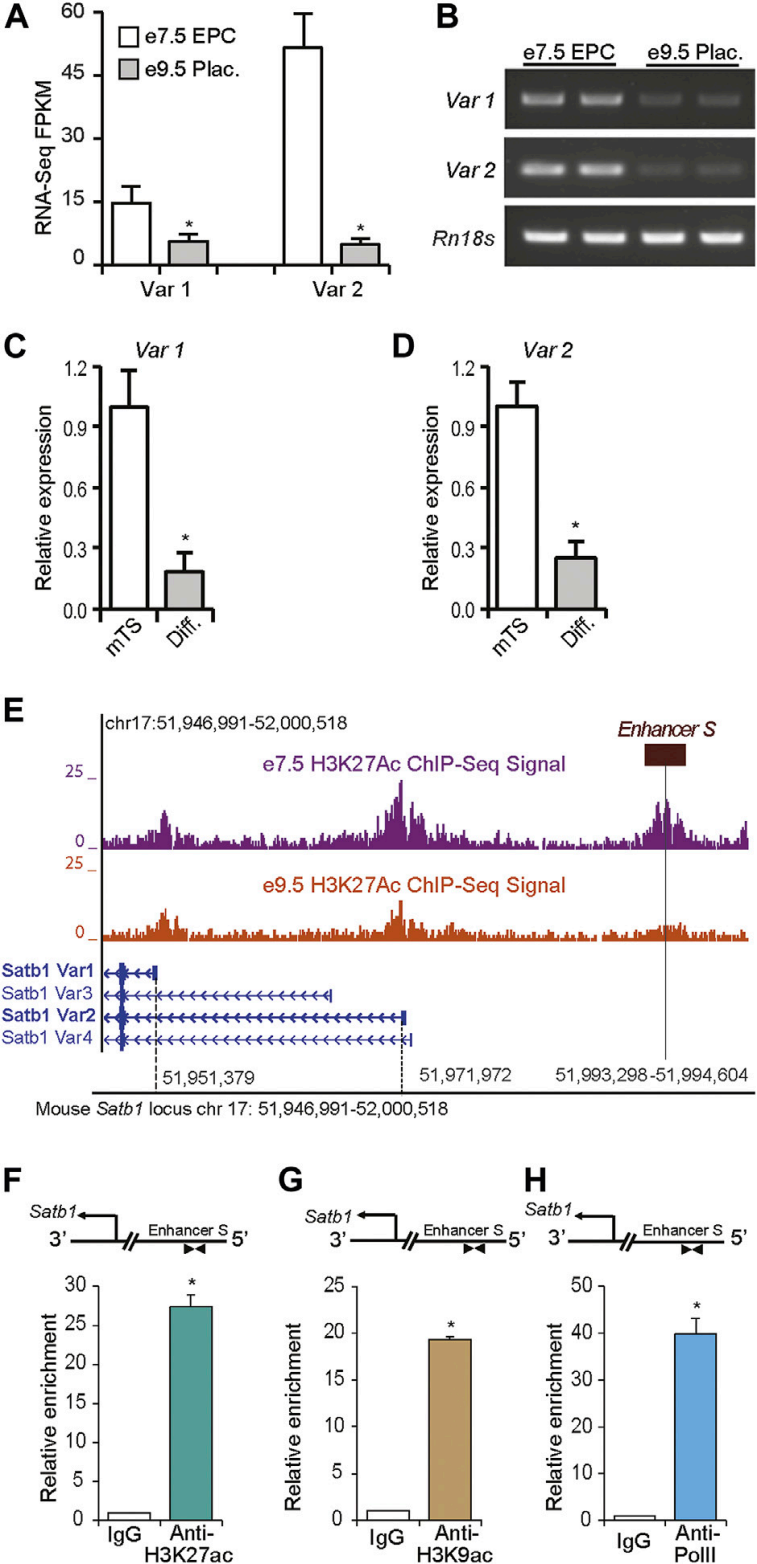
A long-distance enhancer regulates *Satb1* expression in mouse TS cells. Expression of *Satb1* transcript variants 1 and 2 in mouse placenta and mouse TS cells were detected by RNA-seq **(A)** (Tuteja et al., 2016), RT-PCR **(B)** and RT-qPCR **(C–D)** analysis. The expression of both transcript variants was markedly reduced in e9.5 placenta compared to that in e7.5 EPC **(A,B)**. A similar reduction in *Satb1* expression was also observed in differentiated TS cells **(C,D)**. Such reductions in *Satb1* expression levels correlated with the epigenetic marks of the active chromatin state in *Satb1* promoters and an e7.5-specific distal enhancer (*enhancer S*) ∼ 21 kbp upstream of the variant 2 transcription start site, as detected by H3K27ac ChIP-seq (Tuteja et al., 2016) **(E)**. Mouse TS cells were positive for enrichment of H3K27ac, and H3K9ac at this potential enhancer site **(F,G)**. Enriched Pol II binding at the enhancer was also detected by ChIP **(H)**. ChIP-qPCR primers located in the enhancer region are shown schematically in **(F–H)**. The primer sequences are mentioned in Supplementary Table S4. RNA-seq FPKM, RT-qPCR, and ChIP-qPCR data are expressed as mean ± S.D. *p < 0.05 (*n* = 3). EPC, ectoplacental cone; Plac., Placenta.

Reference sequences of four different transcript variants of mouse *Satb1* mRNA have been reported and validated (Supplementary FiguresS2A, S2B). RT-PCR analyses suggested that the first exon in each variant is transcribed from alternative transcriptional start sites over a span of 21 kbp of genomic DNA (Figures 2A–C; Supplementary FigureS2A,B). Only transcripts of variants 1 and 2 were detected in mouse trophoblast cells of e7.5 ectoplacental cones (EPCs) (Figures 2C,3B). ChIP-sequencing (ChIP-seq) analyses for H3K27ac in mouse e7.5 EPCs demonstrated the presence of this transcriptional activation mark in the proximal promoters of both transcript variants (Figure 2D). Both promoters also contained CpG islands (Figure 2D).

The variant 2 promoter in mouse TS cells was also examined for the active histone marks, H3K27 acetylation and H3K4 methylation, as well as RNA polymerase II (Pol II) occupancy. ChIP-qPCR was performed with primers designed against the Satb1 proximal promoter region in three different immunoprecipitated chromatin samples pulled down with antibodies against H3K27ac, H3K4me3, Pol II, positive, and negative controls (Figures 2E–G; SupplementaryFigureS2C–F). Our ChIP-qPCR assay results confirm the early placental ChIP-seq data for H3K27ac (Tuteja et al., 2016) (Figure 2E), exhibiting enrichment of this active histone mark in the proximal promoter of this variant in mouse TS cells. The promoter also showed enriched marks of H3K4me3 (Figure 2F), and RNA polymerase II (Pol II) occupancy (Figure 2G) as analyzed by the ChIP-qPCR, while the positive and negative control primer sets exhibited the expected no enrichment of histone marks or Pol II binding (Supplementary FigureS2C–F).

### 3.3 Identification of a distant-acting enhancer for the *Satb1* gene

RNA-Seq and RT-PCR data indicate that the expression of both transcript variants of mouse *Satb1* were markedly reduced, *in vivo*, in e9.5 placentas compared to e7.5 EPCs (Figures 3A,B). Similar reductions in *Satb1* expression were detected with RT-qPCR during differentiation of mouse TS cells, *in vitro*; expression of both variant 1 and variant 2 were significantly decreased (Figures 3C,D). Such reductions in expression correlated well with the changes in H3K27ac activity within a potential *cis-*acting enhancer region (*enhancer S*) approximately 21 kbp upstream of the *Satb1* variant 2 promoter (Figure 3E). When ChIP assays were performed on mouse TS cells, we also detected enriched histone marks, H3K27ac and H3K9ac, as well as enrichment of Pol II binding in the enhancer region (Figures 3F–H). We termed this distant-acting *cis* enhancer, *enhancer S*, a potential enhancer of *Satb1*.

### 3.4 Distant-acting *enhancer S* is required for maintaining *Satb1* expression in TS cells

**FIGURE 4:**
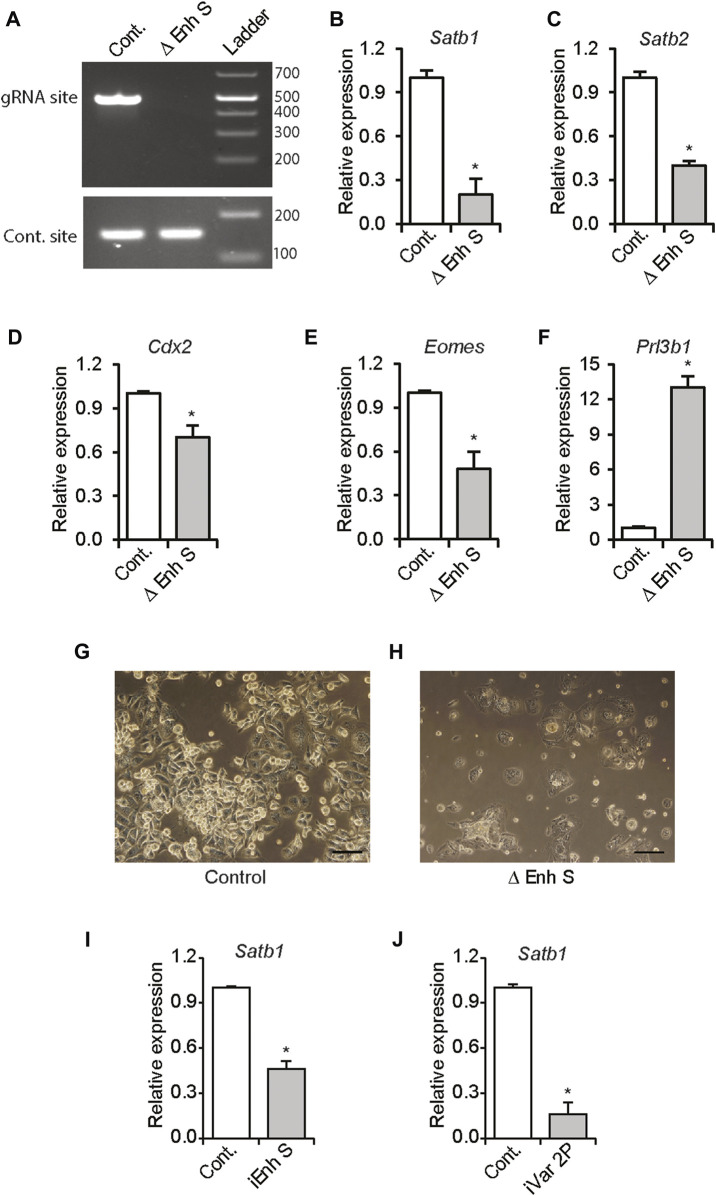
*Enhancer S* is required for *Satb1* expression. Rcho1 rat trophoblast cells were transfected with Cas9 and control or targeted gRNA expression constructs. Stably transfected cells were selected and assessed for targeted deletion of the enhancer. Applying the CRISPR/Cas9 system resulted in the deletion of the gRNA targeted site in *enhancer S* (Δ Enh S) **(A)**, decreased *Satb1* and *Satb2* expression **(B,C)** and caused differentiation of Rcho1 cells, characterized by deceased expression of *Cdx2* and *Eomes*, and increased expression of *Prl3b1* **(D–H)**. The requirement of *enhancer S* was further confirmed by transient transfection of dCas9-KRAB and the enhancer targeted gRNAs (iEnh S) **(I)**. Transfection of gRNAs targeted to the variant 2 promoter (iVar2P) was used as positive control **(J)**. RT-qPCR data are expressed as the means ± S.D. *p < 0.05 (*n* = 3).

Using the CRISPR/Cas9 methodology, we investigated whether the distant enhancer was required for maintaining *Satb1* expression in TS cells. Transfection of expression vectors encoding Cas9, and the enhancer targeted gRNAs resulted in deletion of *enhancer S* in Rcho1 rat TS cells (Figure 4A). Deletion of the enhancer caused dramatic reductions in *Satb1* and *Satb2* expression (Figures 4B,C), which corresponded with an associated induction of premature differentiation in Rcho1 cells maintained in a proliferating culture condition (Figures 4D–H). Premature differentiation of Rcho1 cells was identified by the reduction of stem markers *Cdx2* and *Eomes,* and an increase of the differentiation marker *Prl3b1* (Figures 4D–F). To determine whether the reduction in *Satb1* expression was due to induction of differentiation or disruption of *enhancer S*, we further investigated its requirement using CRISPR interference. Transfection of dCas9-repressor (dCas9-KRAB) and gRNAs targeted to the enhancer sequence also markedly reduced *Satb1* expression (Figure 4I). CRISPR interference of *enhancer S* reduced *Satb1* expression in the same way as interference of the variant 2 promoter in Rcho1 TS cells (Figure 4J).

### 3.5 *Enhancer S* loops into the proximal promoter to regulate *Satb1* expression

**FIGURE 5:**
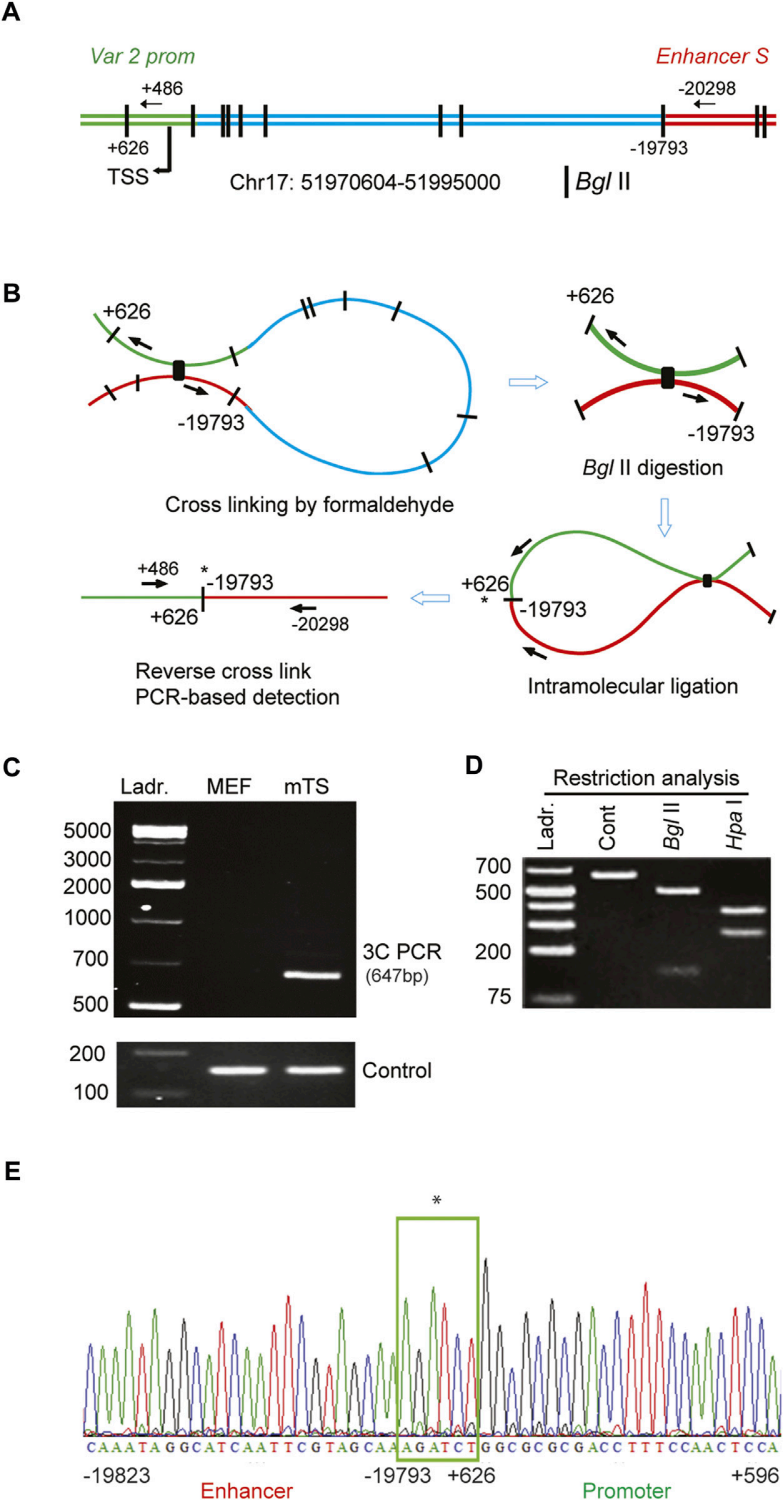
*Enhancer S* loops into the *Satb1* promoter in mouse TS cells. **(A)** Schematic diagram of the mouse *Satb1* locus showing the variant 2 promoter (var 2 prom), transcription start site (TSS), *Bgl* II restriction sites, and 3C PCR primer positions. **(B)** Representation of the major steps of 3C PCR-based detection of the looping and interaction of *enhancer S* with the *Satb1* var 2 promoter. 3C PCR detected a physical interaction of the enhancer with the *Satb1* promoter in mouse TS (mTS) cells but not in mouse embryonic fibroblast (MEF) cells **(C)**. The 3C PCR product (648 bp) was confirmed by restriction analyses **(D)** as well as DNA sequencing **(E)**. *indicates DNA ligation site. Ladr., DNA ladder.

We examined the molecular mechanism of how the distant-acting *enhancer S* regulated *Satb1* expression. Involvement of chromatin looping that can bring the enhancer into proximity with the promoter was tested by 3C in mouse TS cells (Figures 5A,B). A looping interaction between enhancer S and the Satb1 variant 2 promoter was detected by 3C-PCR in mouse TS cells, but not in MEFs (Figure 5C). Restriction analyses (Figure 5D) and DNA sequencing (Figure 5E) confirmed that the 3C-PCR captured and amplified a ligation between the distant-acting enhancer S and the Satb1 variant 2 promoter.

### 3.6 Transcriptional regulation of *enhancer S* in TS cells

**FIGURE 6:**
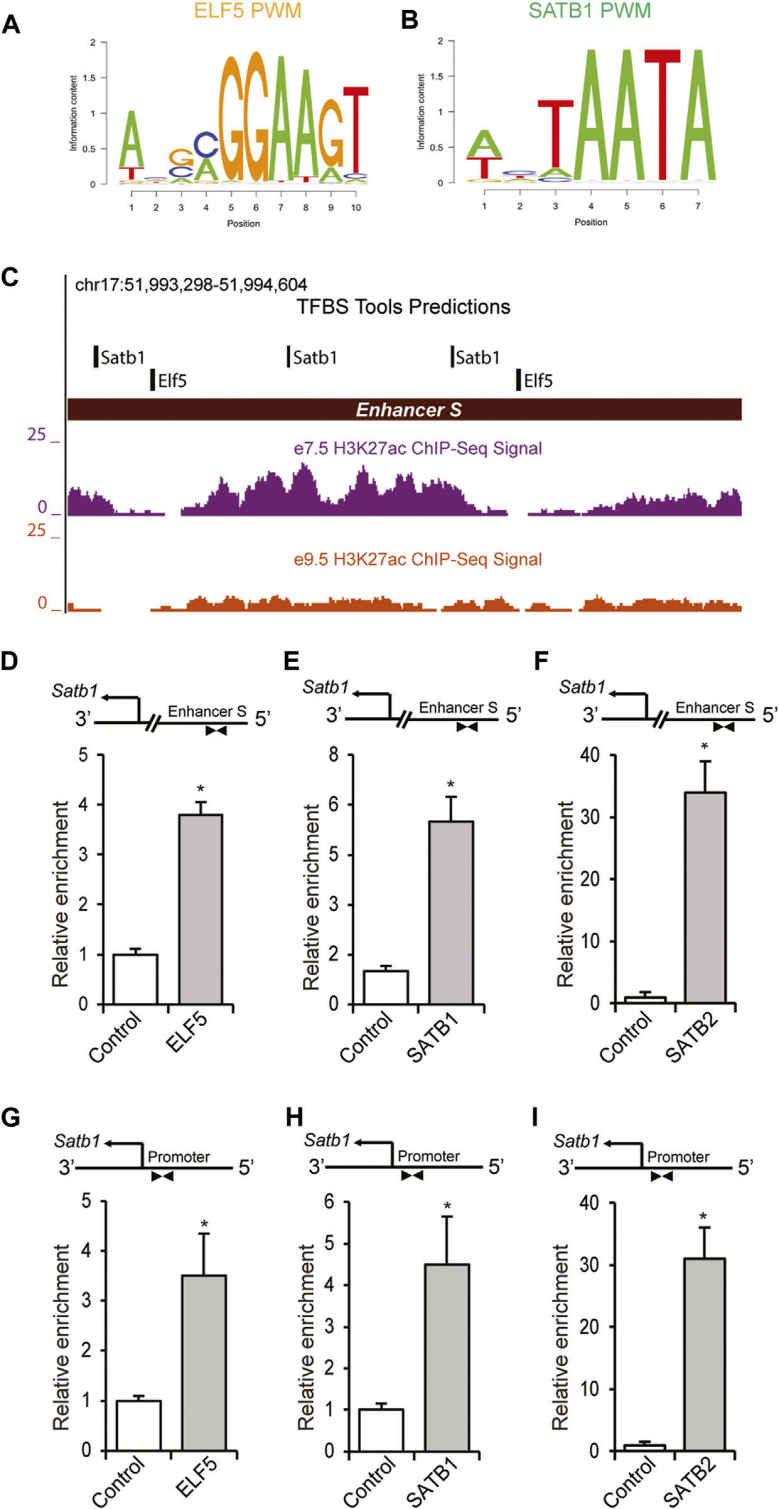
ELF5 and SATB proteins bind within the *enhancer S* in mouse TS cells. **(A,B)** PWMs of ELF5 and SATB1 used for scanning the *enhancer S* sequence (Chr17: 51993298–51994604) using TFBSTools. **(C)** Transcription factor binding site analysis by TFBSTools predicted the presence of two ELF5 binding sites near SATB1 binding sites within *enhancer S.* ChIP assays also demonstrated significant enrichment of ELF5, SATB1 and SATB2 in the enhancer locus of mTS cells **(D–F)**. **(G–I)**, In addition to the enhancer region, binding of ELF5, SATB1, and SATB2 was detected in the *Satb1* variant 2 promoter in mTS cells. ChIP-qPCR data are expressed as the means ± S.D. *p < 0.05 (*n* = 3).

Scanning position weight matrix (PWM) analyses (Figures 6A,B) of *enhancer S* using TFBSTools predicted two ELF5 binding sites in close proximity to the SATB1 binding sites (illustrated in Figure 6C). ChIP assays were performed on mouse TS cells as described in the Section 2. This analysis revealed a marked enrichment of ELF5, SATB1 and SATB2 binding to the *enhancer S* region (Figures 6D–F), as well as in the *Satb1* variant 2 promoter (Figures 6G–I).

**FIGURE 7:**
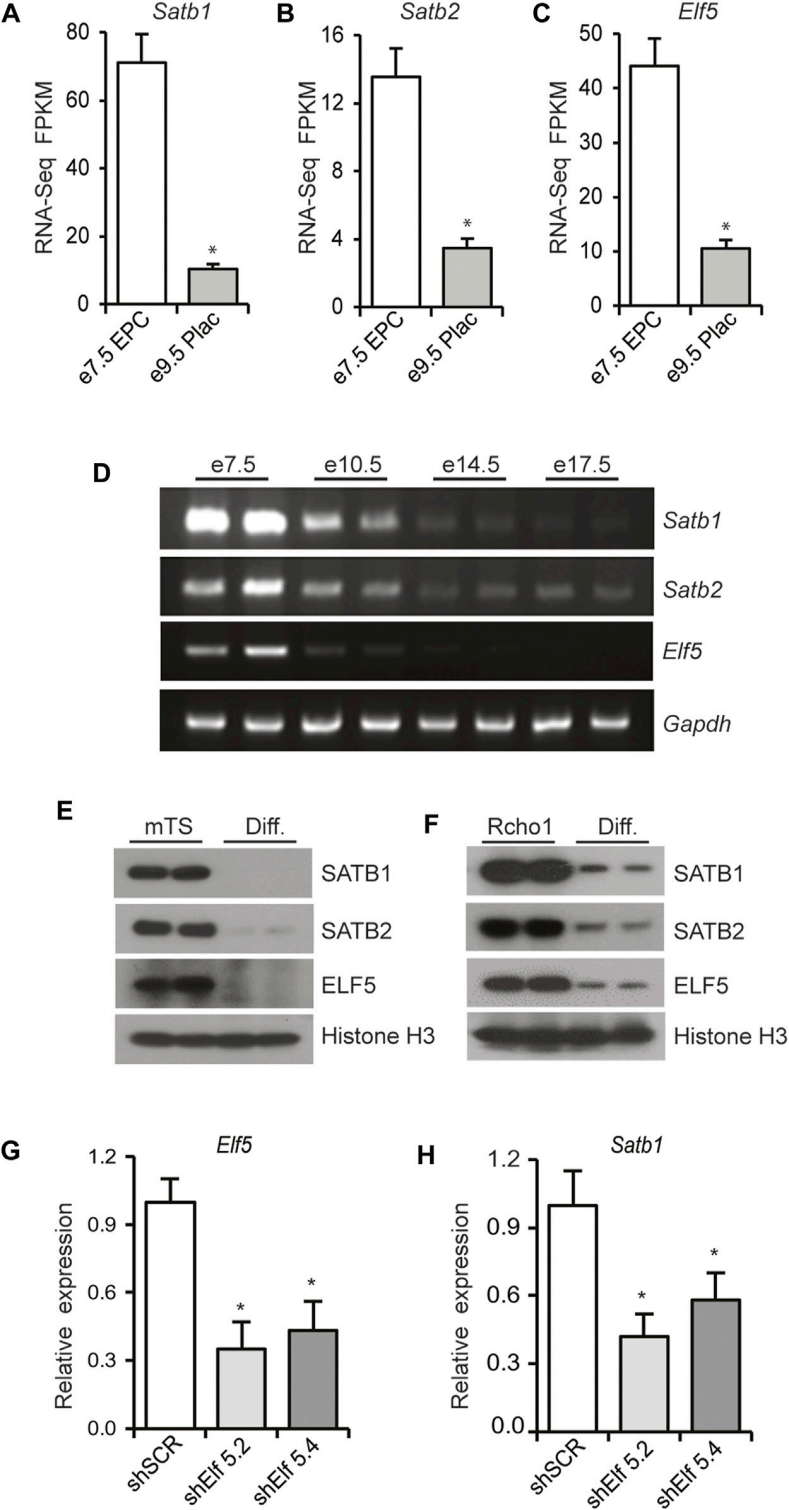
ELF5 regulates *Satb1* expression in TS cells. **(A–C)** RNA-seq analysis demonstrated that expression of *Satb1*, *Satb2*, and *Elf5* is dramatically reduced in mouse e9.5 placentas compared to e7.5 EPCs. Similar findings were observed in RT-PCR analyses of mouse placenta samples collected during the progression of gestation **(D)**. Both mouse TS cells and Rcho1 rat TS cells exhibited similar reductions in SATB1, SATB2 and ELF5 proteins upon induction of differentiation **(E,F)**. In G, Rcho1 rat TS cells were stably transduced *via* lentivirus, with either control or *Elf5* shRNAs, 5.2 or 5.4 as described in Section 2. ShRNA mediated knockdown of *Elf5* **(G)** significantly reduced *Satb1* mRNA expression **(H)**, suggesting a role of Elf5 in transcriptional regulation of *Satb1*. RNA-Seq FPKM and RT-qPCR data are expressed as the means ± S.D. *p < 0.05 (*n* = 3).

RNAseq analysis of cells from e7.5 EPC demonstrated significantly higher expression of *Satb1, Satb2*, and *Elf5* mRNA compared to that expressed in e9.5 placenta (Figures 7A–C). In addition, placenta collected during the progression of gestation exhibited progressive reductions in the expression of these three genes as determined by RT-PCR, with the highest levels of expression observed in e7.5 EPC (Figure 7D). Cultured mTS cells and Rcho1 cells were differentiated, *in vitro* (as described in Section 2). Western blot analyses revealed that in both cell lines, the differentiated cells expressed significant reductions in SATB1, SATB2, and ELF5 proteins, compared to the undifferentiated cells (Figures 7E,F). Collectively, these data demonstrate that SATB and ELF5 mRNA and proteins exhibit trophoblast stem-state specific differential expression. We next analyzed the role of ELF5 in regulation of *Satb1* mRNA expression by shRNA mediated knockdown of *Elf5* in Rcho1 TS cells. Knockdown of ELF5 significantly downregulated the expression of Satb1 (Figures 7G,H), consistent with a role of ELF5 in regulating transcription of Satb1.

**FIGURE 8:**
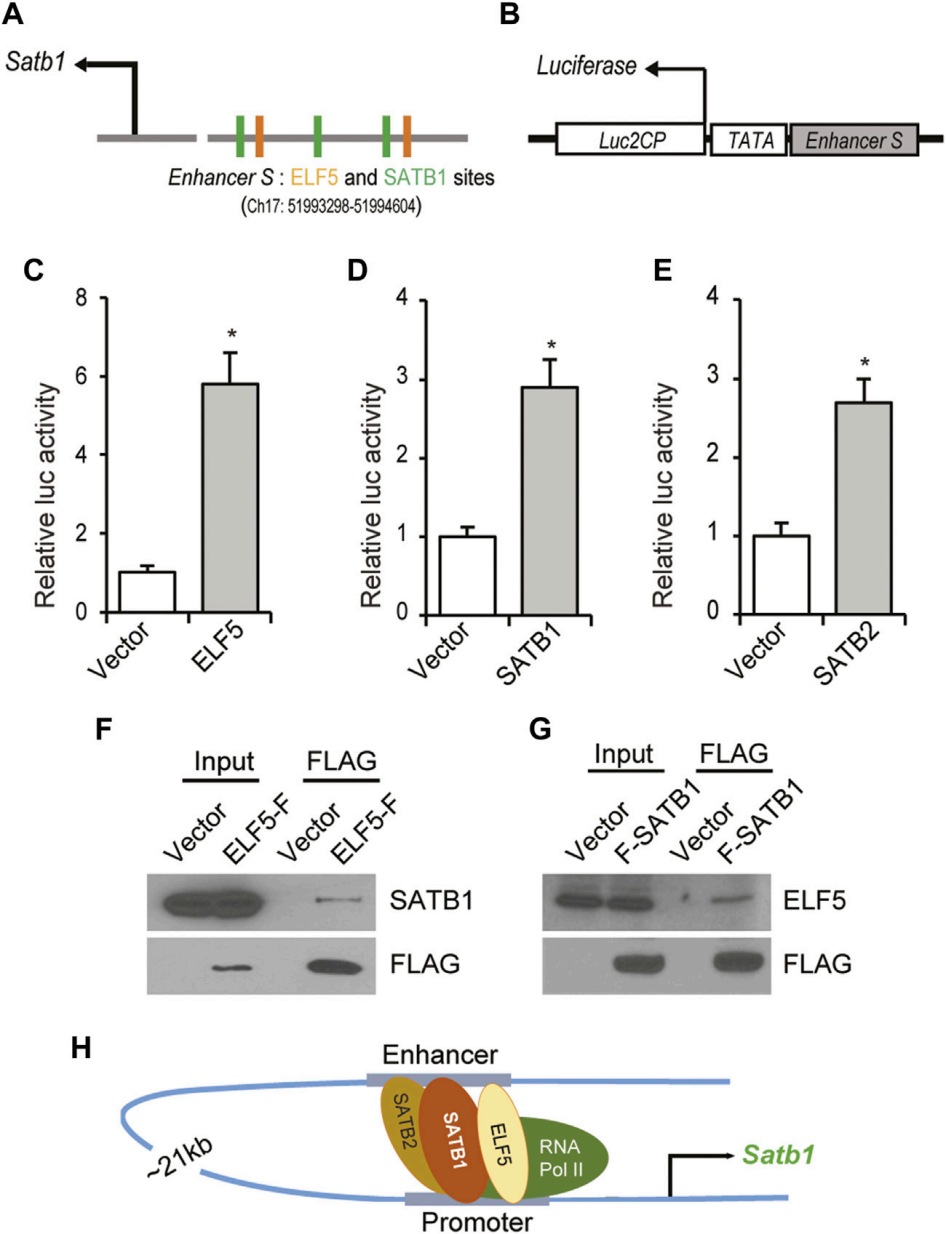
ELF5-SATB1 interaction within the *enhancer S*. **(A)** Schematic diagram showing the TFBSTools-detected two ELF5 binding sites near SATB1 motifs in mouse Satb1 enhancer sequence. **(B)** An enhancer-reporter construct was prepared by cloning 1.5 Kb of *enhancer S* upstream of a minimal TATA promoter within the *Luc2CP* firefly luciferase vector. **(C–E)**, Ectopic expression of ELF5, SATB1 or SATB2 in Rcho1 rat TS cells significantly upregulated the promoter-reporter activity. Furthermore, co-immunoprecipitation of either ELF5 or SATB1 with Rcho1 nuclear proteins demonstrated that SATB1 interacts with ELF5 in trophoblast cells **(F,G)**. Taken together, we propose a model of ELF5-SATB interaction that regulates *Satb1* expression in the trophoblast stem-state **(H)**. Luciferase assay data are expressed as mean ± S.D. *, *p* < 0.05 (*n* = 3). ELF5-F, ELF5 with C-terminal FLAG tag; F-SATB1, SATB1 with N-terminal FLAG Tag.

To assess the role of these transcriptional regulators on *enhancer S*, a reporter construct was prepared by cloning *enhancer S* (Figure 8A) upstream of a minimal TATA promoter into the pGL4.25 [luc2CP/minP] firefly luciferase vector (Figure 8B). Cotransfection of the enhancer-reporter and expression vectors for ELF5, SATB1 or SATB2 into Rcho1 rat TS cells significantly upregulated reporter activity (Figures 8C–E). Furthermore, co-immunoprecipitation of either ELF5 or SATB1 with Rcho1 nuclear proteins detected an interaction between ELF5 and SATB1 (Figures 8F,G). Taken together, we propose a model of ELF5-SATB1 interaction that regulates Satb1 expression in the trophoblast stem-state (Figure 8H).

## 4 Discussion

SATB proteins play essential regulatory roles in a range of stem cells (Savarese et al., 2009; Asanoma et al., 2012; Will et al., 2013; Goolam & Zernicka-Goetz, 2017). During early embryonic development, ES, TS, and XEN cells are the three stem cell lineages that give rise to the embryo proper, placenta, and yolk sac, respectively. Among these three stem cell lineages, only TS cells exhibit robust expression of SATB1 (Figure 1). Expression of *Satb1* was induced during the reprogramming of mouse ES cells or human ES cells to TS cells (Supplementary FigureS1), which was also reported in previous studies (Ralston et al., 2010; Amita et al., 2013). Such induction of *Satb1* expression during reprogramming of ES cells to trophoblast fate indicates that trophoblast-specific cell signaling facilitates the expression. It has recently been shown that disruption of FGF4 signaling, which is essential for TS cell maintenance, may impact *Satb1* expression in mouse preimplantation embryos (Goolam & Zernicka-Goetz, 2017).

Expression of *Satb1* in trophoblast cells has been reported to be stem-state-specific both *in vivo* and *in vitro* (Kent et al., 2010; Asanoma et al., 2012). Differential expression of *Satb*1 in the trophoblast stem-state suggests an important role for stem-specific transcriptional regulators controlling its expression. However, the upstream transcription factors that regulate stem-state specific expression of *Satb1* in trophoblast cells are still unknown.

*Satb1* is an essential regulator of T cell differentiation and FoxP3 plays an important role in transcriptional repression of *Satb1* in regulatory T cells (Beyer et al., 2011). *Satb1* is also an important chromatin regulator in the *epidermis*, where p63 is essential for maintaining *Satb1* gene expression (Fessing et al., 2011). However, based on available GEO data (GSE12999 and GSE21938) expression of both FoxP3 and p63 is very low in TS cells, and they do not show any change in expression with induction of differentiation (Kent et al., 2010; Ralston et al., 2010). These findings suggest that regulation of *Satb1* in trophoblast cells is different from T cells and *epidermis*. To explore the trophoblast-specific *Satb1* regulation, we identified *Satb1* promoters in TS cells. In contrast to T cells that express all four *Satb1* variants, only variant 1 and 2 transcripts were detected in trophoblast cells, with variant 2 being predominant. These proximal promoters were enriched with H3K27ac and H3K4me3, which are marks of active promoters (Consortium, 2012). Presence of CpG islands within the promoters of Satb1 suggests its potential role as a master developmental regulator (Ponger et al., 2001; Tanay et al., 2007; Vavouri and Lehner, 2012).

An enhancer region ∼21 kbp upstream of the Satb1 variant 2 promoter was identified based on active histone marks (Tuteja et al., 2016). Changes in H3K27ac activity in this enhancer region (*enhancer S*) correlated with *Satb1* expression levels in trophoblast cells. Requirement of the enhancer for *Satb1* expression was demonstrated by CRISPR/CAS9 mediated targeted deletion of this region. Targeted deletion of *enhancer S* reduced *Satb1* expression, which caused differentiation of Rcho1 TS cells maintained in proliferating media. This observation is in line with our previous report, where we showed induction of TS cell differentiation following *Satb1* knockdown (Asanoma et al., 2012). However, trophoblast differentiation due to other reasons can also lead to inhibition of *Satb1* expression. We utilized a transient induction of CRISPR interference to avoid the effect of cell differentiation. CRISPR interference provided direct evidence for the importance of this enhancer in regulating *Satb1* expression in TS cells.

Long-range chromatin interactions can occur intrachromosomally or interchromosomally (Deng & Blobel, 2010; Dean, 2011). Intrachromosomal interactions have been reported between promoters and enhancers located far away from each other (Deng & Blobel, 2010; Dean, 2011). In this study, we detected a chromatin loop of the cis-acting enhancer to the *Satb1* variant 2 promoter across a 21 kbp distance. Bioinformatic analyses indicated potential ELF5 binding sites near SATB1 binding sites within the *enhancer S* region. ChIP and reporter assays demonstrated that ELF5 and SATB homeobox proteins bind to *enhancer S* and had a stimulatory effect on the enhancer activity. Binding of ELF5, SATB1 and SATB2 was also detected within the proximal promoter (Figures 8A, 8C–8E). These findings suggest that the looping interaction between the enhancer and the proximal promoter in mouse TS cells was mediated by SATB proteins in association with ELF5. In TS cells, ELF5 can interact with other transcription factors and act as a molecular switch regulating cell differentiation (Latos et al., 2015). SATB1 and SATB2 can also form heterodimers to regulate gene expression (Asanoma et al., 2012; Zhou et al., 2012). It is also well-known that SATB1 can mediate long-range chromatin interactions for gene regulation (Yasui et al., 2002; Gong et al., 2011; Yang et al., 2015). Thus, ELF5 interaction with SATB1 to regulate gene expression over a long distance is a plausible mechanism of the transcriptional regulation of *Satb1*.

Trophoblast stem-specific *Satb1* expression suggests that differentially expressed stem-factors may play a crucial role in regulation of *Satb1*. Indeed, SATB proteins as well as ELF5 exhibited trophoblast stem-specific differential expression both *in vivo* and *in vitro* (Figures 7A–F). We identified that ELF5 plays an important role in regulating *Satb1* expression (Figures 7G,H). Developmentally, expression of ELF5 is restricted to the trophoblast lineage and creates a positive feedback loop with other TS cell determinants (Ng et al., 2008). We previously demonstrated that SATB proteins contribute to the TS cell stem-state by sustaining the expression of TS factors (Asanoma et al., 2012). Therefore, it is likely that SATB proteins interact with ELF5 in TS cells to augment a positive feedback loop to maintain the trophoblast stem-state.

## Data Availability

The datasets presented in this study can be found in online repositories. The names of the repository/repositories and accession number(s) can be found below: https://www.ncbi.nlm.nih.gov/geo/, GSE65808; https://www.ncbi.nlm.nih.gov/geo/, GSE65807.

## Supplementary material

SUPPLEMENTARY FIGURE S1 Induction of Satb1 expression following reprogramming of mouse or human ES cells. Expression of Satb1 was induced when mES cells were reprogrammed towards trophoblast lineage following the overexpression of CDX2 **(A)** or GATA3 **(D)** (Ralston et al., 2010). Increased expression of Stab1 in the reprogrammed mES cells was associated with the upregulation of TS markers including Gata3, Elf5, Eomes, and Ascl2 **(A–F)**. BAP (BMP4, A83-01, and PD173074)-induction also induced reprogramming of human ES (hES) cells towards the trophoblast lineage (Amita et al., 2013). Such reprogramming of human ES cells also upregulated SATB1 expression, along with increased expression of CDX2 and ELF5 **(G–I)**. RT-qPCR data are expressed as mean ± S.D. **p*< 0.05 (*n*=3). Cont, Control.

SUPPLEMENTARY FIGURE S2 Satb1 transcript variants and control experiments for ChIP assays. **(A)** Schematic diagram showing the reference 5’ sequences of four different transcript variants of mouse Satb1. **(B)** The accession numbers, noncoding variant specific first exons, common second exons, coding sequences (CDS), and the transcription start sites on mouse chromosome 17 are presented in a tabulated form. Mouse positive and negative control primer sets were used for validating the ChIP assays (Supplementary Figure S2). ChIP-qPCR data are expressed as mean ± S.D. **p* < 0.05 (*n*=3). NC, Negative Control Primer Set; PC, Positive Control Primer Set.

## References

[B1] Alcamo E. A. Chirivella L. Dautzenberg M. Dobreva G. Farinas I. Grosschedl R. (2008). Satb2 regulates callosal projection neuron identity in the developing cerebral cortex. Neuron 57 (3), 364–377. 10.1016/j.neuron.2007.12.012 18255030

[B2] Alvarez J. D. Yasui D. H. Niida H. Joh T. Loh D. Y. Kohwi-Shigematsu T. (2000). The MAR-binding protein SATB1 orchestrates temporal and spatial expression of multiple genes during T-cell development. Genes Dev. 14 (5), 521–535. 10.1101/gad.14.5.521 10716941 PMC316425

[B3] Amita M. Adachi K. Alexenko A. P. Sinha S. Schust D. J. Schulz L. C. (2013). Complete and unidirectional conversion of human embryonic stem cells to trophoblast by BMP4. Proc. Natl. Acad. Sci. U. S. A. 110 (13), E1212–E1221. 10.1073/pnas.1303094110 23493551 PMC3612666

[B4] Asanoma K. Kubota K. Chakraborty D. Renaud S. J. Wake N. Fukushima K. (2012). SATB homeobox proteins regulate trophoblast stem cell renewal and differentiation. J. Biol. Chem. 287 (3), 2257–2268. 10.1074/jbc.M111.287128 22123820 PMC3265903

[B5] Beyer M. Thabet Y. Muller R. U. Sadlon T. Classen S. Lahl K. (2011). Repression of the genome organizer SATB1 in regulatory T cells is required for suppressive function and inhibition of effector differentiation. Nat. Immunol. 12 (9), 898–907. 10.1038/ni.2084 21841785 PMC3669688

[B6] Britanova O. de Juan Romero C. Cheung A. Kwan K. Y. Schwark M. Gyorgy A. (2008). Satb2 is a postmitotic determinant for upper-layer neuron specification in the neocortex. Neuron 57 (3), 378–392. 10.1016/j.neuron.2007.12.028 18255031

[B7] Cai S. Han H. J. Kohwi-Shigematsu T. (2003). Tissue-specific nuclear architecture and gene expression regulated by SATB1. Nat. Genet. 34 (1), 42–51. 10.1038/ng1146 12692553

[B8] Cai S. Lee C. C. Kohwi-Shigematsu T. (2006). SATB1 packages densely looped, transcriptionally active chromatin for coordinated expression of cytokine genes. Nat. Genet. 38 (11), 1278–1288. 10.1038/ng1913 17057718

[B9] Chuong E. B. Rumi M. A. Soares M. J. Baker J. C. (2013). Endogenous retroviruses function as species-specific enhancer elements in the placenta. Nat. Genet. 45 (3), 325–329. 10.1038/ng.2553 23396136 PMC3789077

[B10] Cockburn K. Rossant J. (2010). Making the blastocyst: Lessons from the mouse. J. Clin. Inves. 120 (4), 995–1003. 10.1172/JCI41229 PMC2846056 20364097

[B11] Consortium E. P. (2012). An integrated encyclopedia of DNA elements in the human genome. Nature 489 (7414), 57–74. 10.1038/nature11247 22955616 PMC3439153

[B12] Dale R. K. Pedersen B. S. Quinlan A. R. (2011). Pybedtools: A flexible Python library for manipulating genomic datasets and annotations. Bioinformatics 27 (24), 3423–3424. 10.1093/bioinformatics/btr539 21949271 PMC3232365

[B13] Dean A. (2011). In the loop: Long range chromatin interactions and gene regulation. Brief. Funct. Genomics 10 (1), 3–10. 10.1093/bfgp/elq033 21258045 PMC3040559

[B14] Dekker J. Rippe K. Dekker M. Kleckner N. (2002). Capturing chromosome conformation. Science 295 (5558), 1306–1311. 10.1126/science.1067799 11847345

[B15] Deng W. Blobel G. A. (2010). Do chromatin loops provide epigenetic gene expression states? Curr. Opin. Genet. Dev. 20 (5), 548–554. 10.1016/j.gde.2010.06.007 20598523 PMC2943049

[B16] Dickinson L. A. Dickinson C. D. Kohwi-Shigematsu T. (1997). An atypical homeodomain in SATB1 promotes specific recognition of the key structural element in a matrix attachment region. J. Biol. Chem. 272 (17), 11463–11470. 10.1074/jbc.272.17.11463 9111059

[B17] Dickinson L. A. Joh T. Kohwi Y. Kohwi-Shigematsu T. (1992). A tissue-specific MAR/SAR DNA-binding protein with unusual binding site recognition. Cell 70 (4), 631–645. 10.1016/0092-8674(92)90432-c 1505028

[B18] Dobreva G. Chahrour M. Dautzenberg M. Chirivella L. Kanzler B. Fariñas I. (2006). SATB2 is a multifunctional determinant of craniofacial patterning and osteoblast differentiation. Cell 125 (5), 971–986. 10.1016/j.cell.2006.05.012 16751105

[B19] Dobreva G. Dambacher J. Grosschedl R. (2003). SUMO modification of a novel MAR-binding protein, SATB2, modulates immunoglobulin mu gene expression. Genes Dev. 17 (24), 3048–3061. 10.1101/gad.1153003 14701874 PMC305257

[B20] Fessing M. Y. Mardaryev A. N. Gdula M. R. Sharov A. A. Sharova T. Y. Rapisarda V. (2011). p63 regulates Satb1 to control tissue-specific chromatin remodeling during development of the epidermis. J. Cell Biol. 194 (6), 825–839. 10.1083/jcb.201101148 21930775 PMC3207288

[B21] Gong F. Sun L. Wang Z. Shi J. Li W. Wang S. (2011). The BCL2 gene is regulated by a special AT-rich sequence binding protein 1-mediated long range chromosomal interaction between the promoter and the distal element located within the 3'-UTR. Nucleic Acids Res. 39 (11), 4640–4652. 10.1093/nar/gkr023 21310710 PMC3113567

[B22] Goolam M. Zernicka-Goetz M. (2017). The chromatin modifier Satb1 regulates cell fate through Fgf signalling in the early mouse embryo. Development 144 (8), 1450–1461. 10.1242/dev.144139 28289135 PMC5399666

[B23] Gyorgy A. B. Szemes M. de Juan Romero C. Tarabykin V. Agoston D. V. (2008). SATB2 interacts with chromatin-remodeling molecules in differentiating cortical neurons. Eur. J. Neurosci. 27 (4), 865–873. 10.1111/j.1460-9568.2008.06061.x 18333962

[B24] Kabadi A. M. Gersbach C. A. (2014). Engineering synthetic TALE and CRISPR/Cas9 transcription factors for regulating gene expression. Methods 69 (2), 188–197. 10.1016/j.ymeth.2014.06.014 25010559 PMC4175060

[B25] Kent L. N. Konno T. Soares M. J. (2010). Phosphatidylinositol 3 kinase modulation of trophoblast cell differentiation. BMC Dev. Biol. 10, 97. 10.1186/1471-213X-10-97 20840781 PMC2944162

[B26] Kunath T. Arnaud D. Uy G. D. Okamoto I. Chureau C. Yamanaka Y. (2005). Imprinted X-inactivation in extra-embryonic endoderm cell lines from mouse blastocysts. Development 132 (7), 1649–1661. 10.1242/dev.01715 15753215

[B27] Latos P. A. Sienerth A. R. Murray A. Senner C. E. Muto M. Ikawa M. (2015). Elf5-centered transcription factor hub controls trophoblast stem cell self-renewal and differentiation through stoichiometry-sensitive shifts in target gene networks. Genes Dev. 29 (23), 2435–2448. 10.1101/gad.268821.115 26584622 PMC4691948

[B28] Lee D. S. Rumi M. A. Konno T. Soares M. J. (2009). *In vivo* genetic manipulation of the rat trophoblast cell lineage using lentiviral vector delivery. Genesis 47 (7), 433–439. 10.1002/dvg.20518 19444902 PMC4384464

[B29] Nakayama Y. Mian I. S. Kohwi-Shigematsu T. Ogawa T. (2005). A nuclear targeting determinant for SATB1, a genome organizer in the T cell lineage. Cell Cycle 4 (8), 4099–4106. 10.4161/cc.4.8.1862 15970696

[B30] Ng R. K. Dean W. Dawson C. Lucifero D. Madeja Z. Reik W. (2008). Epigenetic restriction of embryonic cell lineage fate by methylation of Elf5. Nat. Cell Biol. 10 (11), 1280–1290. 10.1038/ncb1786 18836439 PMC2635539

[B31] Notani D. Gottimukkala K. P. Jayani R. S. Limaye A. S. Damle M. V. Mehta S. (2010). Global regulator SATB1 recruits beta-catenin and regulates T(H)2 differentiation in Wnt-dependent manner. PLoS Biol. 8 (1), e1000296. 10.1371/journal.pbio.1000296 20126258 PMC2811152

[B32] Pfeffer P. L. Pearton D. J. (2012). Trophoblast development. Reproduction 143 (3), 231–246. 10.1530/REP-11-0374 22223687

[B33] Ponger L. Duret L. Mouchiroud D. (2001). Determinants of CpG islands: Expression in early embryo and isochore structure. Genome Res. 11 (11), 1854–1860. 10.1101/gr.174501 11691850 PMC311164

[B34] Ralston A. Cox B. J. Nishioka N. Sasaki H. Chea E. Rugg-Gunn P. (2010). Gata3 regulates trophoblast development downstream of Tead4 and in parallel to Cdx2. Development 137 (3), 395–403. 10.1242/dev.038828 20081188

[B35] Roberts R. M. Fisher S. J. (2011). Trophoblast stem cells. Biol. Reprod. 84 (3), 412–421. 10.1095/biolreprod.110.088724 21106963 PMC3043125

[B36] Sahgal N. Canham L. N. Canham B. Soares M. J. (2006). Rcho-1 trophoblast stem cells: A model system for studying trophoblast cell differentiation. Methods Mol. Med. 121, 159–178. 16251742

[B37] Savarese F. Davila A. Nechanitzky R. De La Rosa-Velazquez I. Pereira C. F. Engelke R. (2009). Satb1 and Satb2 regulate embryonic stem cell differentiation and Nanog expression. Genes Dev. 23 (22), 2625–2638. 10.1101/gad.1815709 19933152 PMC2779756

[B38] Tan G. Lenhard B. (2016). TFBSTools: An R/bioconductor package for transcription factor binding site analysis. Bioinformatics 32 (10), 1555–1556. 10.1093/bioinformatics/btw024 26794315 PMC4866524

[B39] Tanaka S. Kunath T. Hadjantonakis A. K. Nagy A. Rossant J. (1998). Promotion of trophoblast stem cell proliferation by FGF4. Science 282 (5396), 2072–2075. 10.1126/science.282.5396.2072 9851926

[B40] Tanay A. O'Donnell A. H. Damelin M. Bestor T. H. (2007). Hyperconserved CpG domains underlie Polycomb-binding sites. Proc. Natl. Acad. Sci. U. S. A. 104 (13), 5521–5526. 10.1073/pnas.0609746104 17376869 PMC1838490

[B41] Tuteja G. Chung T. Bejerano G. (2016). Changes in the enhancer landscape during early placental development uncover a trophoblast invasion gene-enhancer network. Placenta 37, 45–55. 10.1016/j.placenta.2015.11.001 26604129 PMC4707081

[B42] Untergasser A. Nijveen H. Rao X. Bisseling T. Geurts R. Leunissen J. A. (2007). Primer3Plus, an enhanced web interface to Primer3. Nucleic Acids Res. 35, W71–W74. Web Server issue). 10.1093/nar/gkm306 17485472 PMC1933133

[B43] Vavouri T. Lehner B. (2012). Human genes with CpG island promoters have a distinct transcription-associated chromatin organization. Genome Biol. 13 (11), R110. 10.1186/gb-2012-13-11-r110 23186133 PMC3580500

[B44] Wen J. Huang S. Rogers H. Dickinson L. A. Kohwi-Shigematsu T. Noguchi C. T. (2005). SATB1 family protein expressed during early erythroid differentiation modifies globin gene expression. Blood 105 (8), 3330–3339. 10.1182/blood-2004-08-2988 15618465

[B45] Wenger A. M. Clarke S. L. Guturu H. Chen J. Schaar B. T. McLean C. Y. (2013). PRISM offers a comprehensive genomic approach to transcription factor function prediction. Genome Res. 23 (5), 889–904. 10.1101/gr.139071.112 23382538 PMC3638144

[B46] Will B. Vogler T. O. Bartholdy B. Garrett-Bakelman F. Mayer J. Barreyro L. (2013). Satb1 regulates the self-renewal of hematopoietic stem cells by promoting quiescence and repressing differentiation commitment. Nat. Immunol. 14 (5), 437–445. 10.1038/ni.2572 23563689 PMC3633104

[B47] Yang Y. Wang Z. Sun L. Shao L. Yang N. Yu D. (2015). SATB1 mediates long-range chromatin interactions: A dual regulator of anti-apoptotic BCL2 and pro-apoptotic noxa genes. PLoS One 10 (9), e0139170. 10.1371/journal.pone.0139170 26422397 PMC4589335

[B48] Yasui D. Miyano M. Cai S. Varga-Weisz P. Kohwi-Shigematsu T. (2002). SATB1 targets chromatin remodelling to regulate genes over long distances. Nature 419 (6907), 641–645. 10.1038/nature01084 12374985

[B49] Zhou L. Q. Wu J. Wang W. T. Yu W. Zhao G. N. Zhang P. (2012). The AT-rich DNA-binding protein SATB2 promotes expression and physical association of human (G)γ- and (A)γ-globin genes. J. Biol. Chem. 287 (36), 30641–30652. 10.1074/jbc.M112.355271 22825848 PMC3436309

