# Supplementary figures and images for: Transcriptional regulation of Satb1 in mouse trophoblast stem cells
Source: Front Cell Dev Biol. 2022 Dec 14;10:918235. doi: 10.3389/fcell.2022.918235 (PMC9795202; doi:10.3389/fcell.2022.918235)

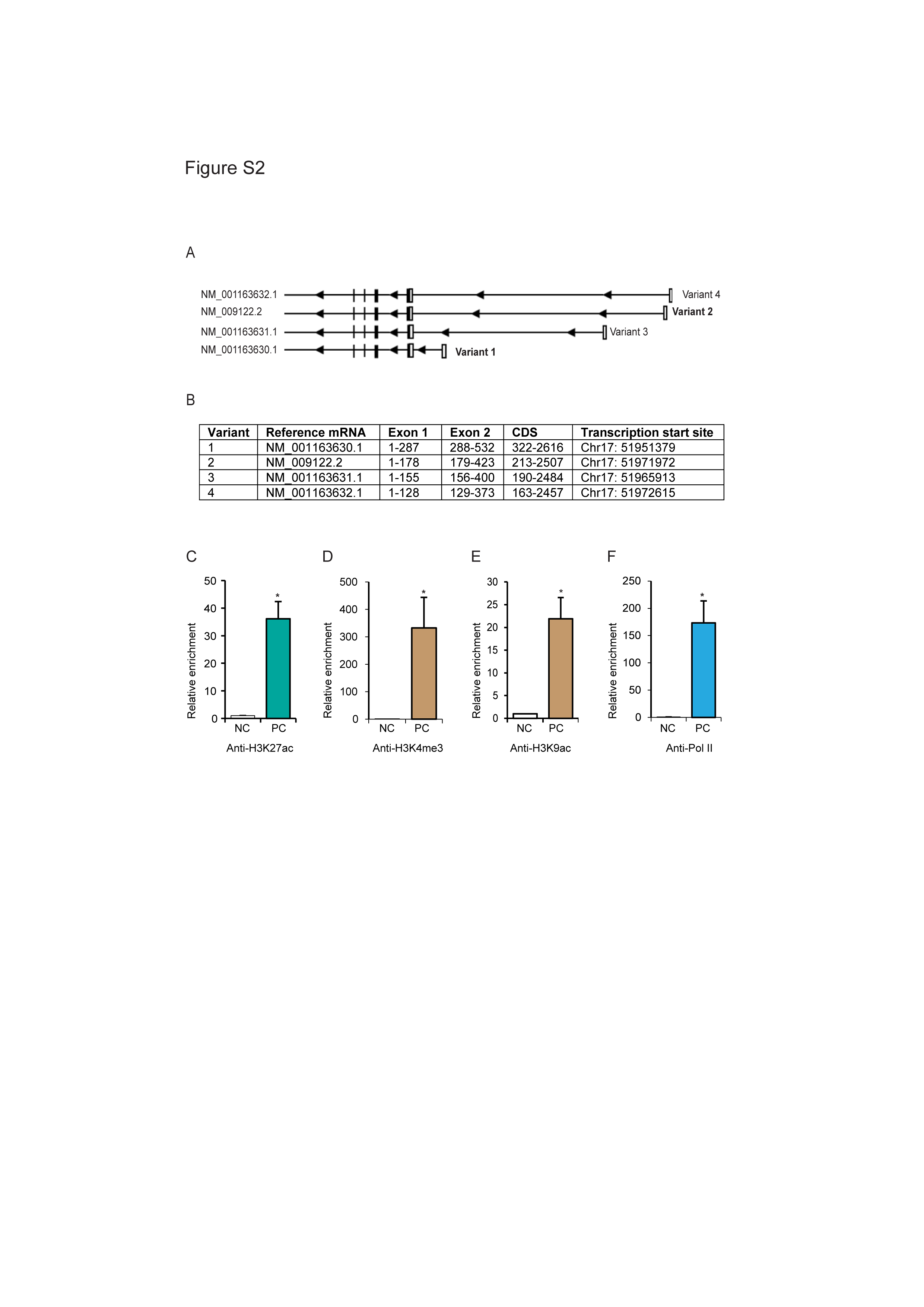

Supplement: Supplementary file 1 [file Image2.TIF]

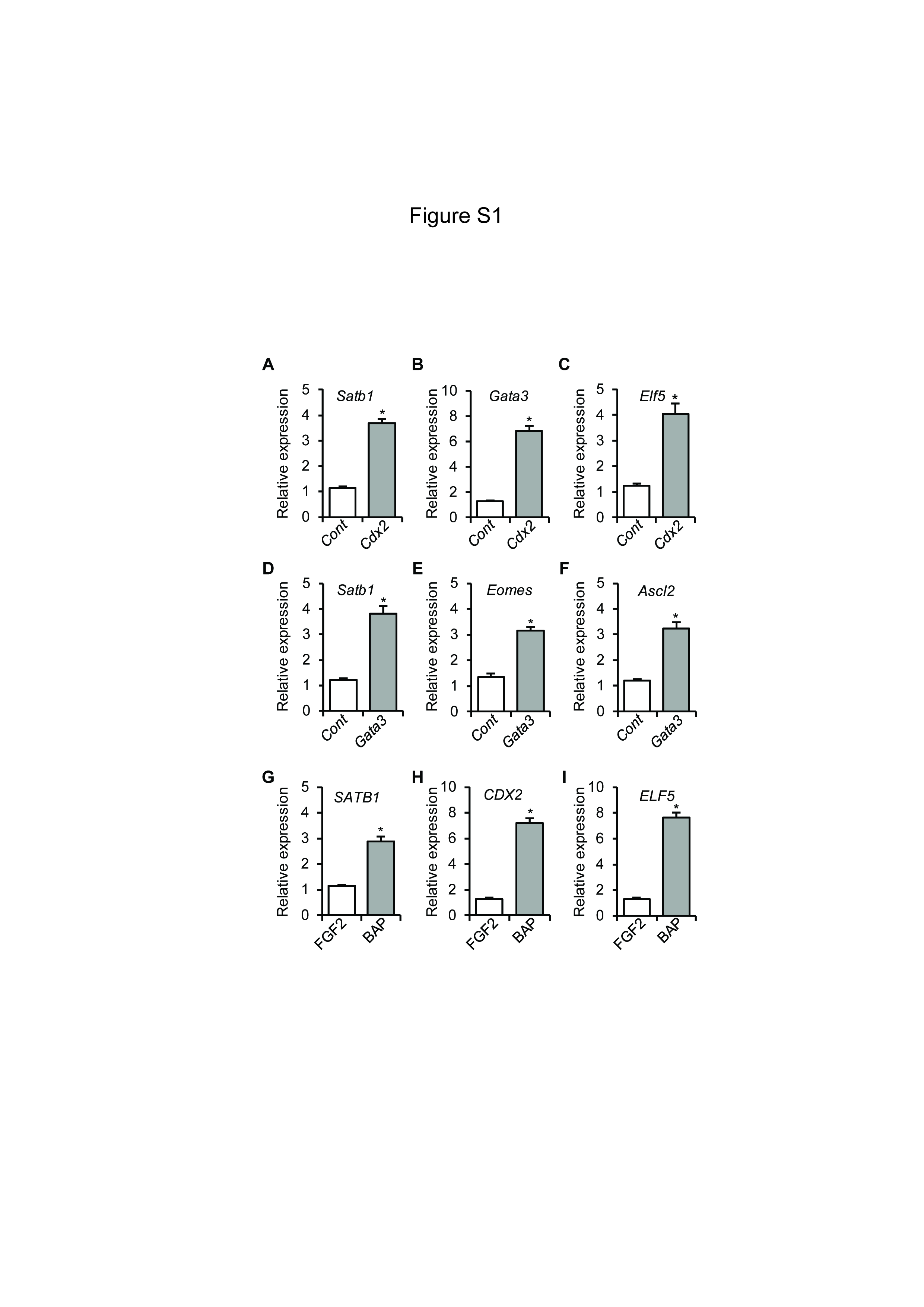

Supplement: Supplementary file 2 [file Image1.TIF]
