# Supplementary material for: Transcriptional regulation of Satb1 in mouse trophoblast stem cells
Source: Front Cell Dev Biol. 2022 Dec 14;10:918235. doi: 10.3389/fcell.2022.918235 (PMC9795202; doi:10.3389/fcell.2022.918235)
Supplement: Supplementary file 3 [file DataSheet1.PDF]

SUPPLEMENTAL TABLES

Table S1. Primers used for conventional RT-PCR analyses

Target	Accession no.	Forward primer	Reverse primer	Amplicon (bp)
Mouse Satb1	NM_009122.2	CTTTGGAGCAGCAAGTTTCC	AGGTTCTCCCACAGGGTCCT	568
Mouse Satb2	NM_139146.2	TACTCCAATCCGAAACCAAG	GTTGTCGGTGTCGAGGTTTT	573
Mouse Elf5	NM_001145813.1	GCTTGAAAACAAGTGGCATC	GTCCGGTGTCCATCAGAGTT	353
Mouse Gapdh	NM_008084.3	ACCACAGTCCATGCCATCAC	TCCACCACCCTGTTGCTGTA	452

Table S2. Primers used for conventional RT-PCR and RT-qPCR analyses of mouse *Satb1* transcript variants

Target	Accession no.	Forward primer	Reverse primer	Amplicon (bp)
Mouse Var1	NM_001163630.1	CCTTCAGGTCTGCTGCTTTT	CACTCCCTGCATCTTTCCAC	351
Mouse Var2	NM_009122.2	AAGTCAAGTTGCCATTAACTTCC	CACTCCCTGCATCTTTCCAC	366
Mouse Var3	NM_001163631.1	GCTAACCTGCCAGAGAGAACTT	CACTCCCTGCATCTTTCCAC	292
Mouse Var4	NM_001163632.1	CCCAGGCAAACAACGACAA	CACTCCCTGCATCTTTCCAC	262
Mouse Rn18s	NR_003278.3	GCAATTATTCCCCATGAACG	GGCCTCACTAAACCATCCAA	123

Table S3. Primers used for RT-qPCR analyses of gene expression

Symbol	Accession no.	Forward primer	Reverse primer	Amplicon (bp)
Human SATB1	NM_001131010.3	GCAGAATTTGTGCTGGTGAG	CCAACCTGGATTAGCCCTTT	119
Human CDX2	NM_001265.6	GTACCCGGACTACGGCGGTT	GCCATTCCAGTCCTCCCGGA	130
Human ELF5	NM_001243080.2	GCCTTGAAAGCCTCCTCTTT	CTGTCTGATGCTCAAAGGCA	169
Mouse Satb1	NM_009122.2	GTGGCAGACATGCTTCAAGA	TACTGTGGTGTGCGACCATT	111
Mouse Gata3	NM_008091.3	GGGTTCGGATGTAAGTCGAG	CCACAGTGGGGTAGAGGTT	90
Mouse Elf5	NM_010125.3	TGCCTTTGAGCATCAGACAG	TACTGGTCGCAGCAGAATTG	108
Mouse Eomes	NM_010136.4	GGTACGGCGTTCAAAACTTC	AGAGGAGGCCGTTGGTCTGT	105
Mouse Ascl2	NM_008554.3	TTTTCGAGGACGCAATAAGC	CACTGCTGCAGGACTCCCTA	120
Rat Satb1	NM_001012129	TGAGAGGGAAAGGAGCTTGA	TGTTCTCTGGCTTCCCATTC	131
Rat Satb2	NM_001109306	CTTCCTCAACCTGCCTGAAG	GTTGTCGGTGTCGAGGTTTT	150

Rat Cdx2	NM_023963.1	CAGGAGGAAAGCTGAGTTGG	TGCTGCTGTTGCAACTTCTT	120
Rat Eomes	XM_017596193.1	CAATGTGTTCGTGGAAGTGG	GTTGGGAGATTCTGGGTGAA	133
Rat Prl3d1	NM_017363.3	TCTTCCGGGAGCTTCTGTTA	GACCAGGCAGGGTAGTCAAA	150
I8SrRNA/ Rn18s (Human, Mouse, and Rat)	NR_145819.1<br>NR_003278.3<br>NR_046237.1	GCAATTATTCCCCATGAACG	GGCCTCACTAAACCATCCAA	123

Table S4. Primers used for ChIP qPCR analyses

**Target**	**Forward primer**	**Reverse primer**	**Amplicon (bp)**
Mouse Enhancer S	AGCAGGTGTGAGCAGCTGAG	GACTGTCCTTCAAGTCTTTCTCA	137
Rat Enhancer S	AGCAGGTGTGAGCAGCTGAG	CCTGTCCTTCAAGTCTTTCTCA	135
Mouse Var 2 prom	GGCCACTGAGAAGTTTGGAT	TGAGTGAGTCCCGCTTCTTT	113
Rat Var 2 prom	GGCTCCCGGGATAGAGAAGTT	GGGTGGAGTTGGAAAGGTC	161

Enhancer S, *Satb1* enhancer; Var 2 prom, *Satb1* variant 2 promoter.

Table S5. gRNAs used for CRISPR- interference and deletion of *enhancer S* and the *Satb1* variant 2 promoter

**Target**	**Sites**	**Forward oligos**	**Reverse oligos**
Rat Enhancer S	*1*	rSatb1*Enhancer S* gRNA-1F<br>CACC GAGAGGCACATCCGGTAAGT	rSatb1 *Enhancer S* gRNA-1R<br>AAAC ACTTACCGGATGTGCCTCTC
Rat Enhancer S	*2*	rSatb1 *Enhancer S* gRNA-2F<br>CACC GCCCTGCAGAACGCACTCTA	rSatb1 *Enhancer S* gRNA-2R<br>AAAC TAGAGTGCGTTCTGCAGGGC
Rat Enhancer S	*3*	rSatb1 *Enhancer S* gRNA-3F<br>CACC GTGGCGAACAGGTGTCTACT	rSatb1 *Enhancer S* gRNA-3R<br>AAAC AGTAGACACCTGTTCGCCAC
Rat Var 2 prom	*1*	rSatb1 Var2 pro gRNA-1F<br>CACC CATCCGAAGTGGGCGTTTAA	rSatb1 Var2 pro gRNA-1R<br>AAAC TTAAACGCCCACTTCGGATG
Rat Var 2 prom	*2*	rSatb1 Var2 pro gRNA-2F<br>CACC GAGCGCGGCGAGCAGCGAGC	rSatb1 Var2 pro gRNA-2R<br>AAAC GCTCGCTGCTCGCCGCGCTC
Rat Var 2 prom	*3*	rSatb1 Var2 pro gRNA-3F<br>CACC GTAGCGCGCGGCCGAGGGGA	rSatb1 Var2 pro gRNA-3R<br>AAAC TCCCCTCGGCCGCGCGCTAC

Color code: Blue- cloning sites; Black: target sequence; rSatb1, rat *Satb1*; Var 2 prom, Variant 2 promoter

Table S6. Primers used for conventional PCR to detect CRISPR/Cas9 mediated enhancer deletion

Target	Forward primer	Reverse primer	Amplicon (bp)
Rat Enhancer S	ACTTACCGGATGTGCCTCTC	TAGAGTGCGTTCTGCAGGGC	506
Control site (Var 2 prom)	GGCTCCCGGGATAGAGAAGTT	GGGTGGAGTTGGAAAGGTC	161

Table S7. PCR Primers used for Chromatin Conformation Capture (3C) analyses

Primer	Target	Location	Target sequence	Amplicon (bp)
-20298Rv	*Mouse Enhancer S*	Chr17: 51992270<br>506 bp from *Bgl* II site	GAGCTGAATGAGCCTGAAAGA	647
+486Rv	Mouse var 2 promoter	Chr17: 51971486<br>141 bp from *Bgl* II site	TGGCCCGCTTTAGAGGACGAAG	
-20020Rv	*Enhancer S* sequencing	Chr17: 51991992	GTTCAATTGGCCAGGGTTATTT	
Control-F	Enhancer		AGCAGGTGTGAGCAGCTGAG	137
Control-R	Enhancer		GACTGTCCTTCAAGTCTTTCTCA	

Table S8. shRNA target site and sequences

shRNA	Target	Target sequence	**shRNA sequence** (cloned into *Age*I and *Ecor*I sites of pLKO.1)
shElf5.2	Rat *Elf5*	ATCAGATCAAACTAGACATTT	accggtATCAGATCAAACTAGACATTTctcgagAAATGTCTAGTTTGA TCTGATTTTTTTGgaattc
shElf5.4	Rat *Elf5*	GCCCTGAGATACTACTATAAA	accggtGCCCTGAGATACTACTATAAActcgagTTTATAGTAGTATCT CAGGGCTTTTTTgaattc
shSCR	None	CCTAAGGTTAAGTCGCCCTC	accggtCCTAAGGTTAAGTCGCCCTCGctcgagCGAGGGCGACTTAA CCTTAGGTTTTTTgaattc

Color code: Blue- cloning sites; Black: sense target; Orange: shRNA loop; Green: antisense target; Red: stop signal for RNA polymerase III
